# Post-traumatic Stress Disorder in Parents Following Their Child’s Single-Event Trauma: A Meta-Analysis of Prevalence Rates and Risk Factor Correlates

**DOI:** 10.1007/s10567-021-00367-z

**Published:** 2021-09-23

**Authors:** Lucy A. Wilcoxon, Richard Meiser-Stedman, Aaron Burgess

**Affiliations:** Department of Clinical Psychology and Psychological Therapies, Norwich Medical School, University of East Anglia, Norwich, NR4 7TJ UK

**Keywords:** Post-traumatic stress disorder, Prevalence, Risk factor, Parents, Children, Predictor

## Abstract

Evidence suggests parents of children who experience a trauma may develop Post-Traumatic Stress Disorder (PTSD), which can have significant consequences for their own and their child’s functioning. As such, identifying the rates and possible correlates for the development of PTSD in parents is of clinical and theoretical importance, and would enhance our understanding of how best to support families in the aftermath of trauma. This meta-analysis of 41 studies (*n* = 4370) estimated the rate of PTSD in parents following their child’s single-incident trauma to be 17.0% (95% CI 14.1–20.0%); when removing samples which were mixed, or not exclusively single-incident traumas the prevalence estimate dropped to 14.4% (95% CI 10.8–18.5%). Pooled effect sizes of 32 potential correlates for parents developing PTSD were also identified. Medium-to-large effects were found for factors relating to the parent’s post-traumatic cognition, psychological functioning and coping strategies alongside child PTSD. Small effects were found for pre-trauma factors, objective trauma-related variables and demographic factors for both parent and child. Results are consistent with cognitive models of PTSD, suggesting peri- and post-trauma factors are likely to play a substantial role in its development. These findings indicate the clinical need for screening parents most vulnerable to adverse post-traumatic reactions within the context of child trauma and tailoring interventions to include the family where necessary.

**Supplementary Information:**

The online version contains supplementary material available at 10.1007/s10567-021-00367-z.

## Introduction

### Background

Psychological reactions to traumatic events have been studied in adults and children for decades, with the diagnosis of Post-Traumatic Stress Disorder (PTSD) being introduced to the 3rd edition of the Diagnostic and Statistical Manual in 1980 (DSM-III; American Psychiatric Association, 1980). Early research into the development of PTSD acknowledged that exposure to trauma alone was not sufficient to explain the complexity of this response (e.g. Yehuda & McFarlane, 1995). Much research recognises the idiosyncratic nature of responses to trauma for both adults and children in which personal demographics, cognitive, behavioural and environmental factors all play a role (Brewin et al., 2000; Cox et al., 2008; Trickey et al., 2012).

Whilst it is recognised that parents are also at risk of developing secondary PTSD following their child’s trauma, whether or not they are involved in the incident themselves (Landolt et al., 2003; Hiller et al., 2016), parental PTSD is less researched compared to adult and child populations. Kazak et al. (2006) present an integrative model of paediatric medical traumatic stress in which they highlight that child trauma exposure impacts the family system much more widely than just the child. They suggest the need for a systemic approach across all trauma types, in which assessing and understanding how trauma affects families as a whole is fundamental. Consistent with the results from meta-analyses of risk factors for the development of PTSD in adults (Brewin et al., 2000) and children (Trickey et al., 2012), the systemic model considers the impact of trauma across three phases; the traumatic incident itself, and any pre-existing factors related to the individual or family; the immediate systemic aftermath of the trauma; and the longer-term systemic psychological effects of the trauma.

### Clinical Importance

Parental PTSD is of particular clinical importance, both for clinicians working in adult and child mental health services, given the impact this has on both parties (Scheeringa & Zeanah, 2001). By nature of the diagnostic criteria, PTSD is a debilitating condition which impacts on an individuals’ general functioning, however, PTSD in parents is also associated with poorer functioning in their children, through higher incidence of child emotional and behavioural problems (Parsons et al., 2018). Detection of early identifiable factors associated with PTSD in parents could, if offered the appropriate treatment, reduce the likelihood of long-term adverse impacts for both parents and children. Therefore, services offering support in the aftermath of child trauma need to have a greater understanding of the commonness of parental PTSD, and the possible role this may play in the aetiology and maintenance of the child’s presenting problems.

Whilst it may be reasonable to hypothesise that the risk factors for developing PTSD in parents may be similar to those outlined for adults (e.g. Brewin et al., 2000; Ozer et al., 2003), this fails to acknowledge the uniqueness and complexity of the parent role. Adult studies have focussed on the development of PTSD in response to a trauma directly experienced by the individual, however, parental PTSD occurs within response to a child’s trauma. The traumatic event may be indirectly experienced and thus traumatic responses must be understood within the context of secondary trauma (Banyard et al., 2001) and considered through the nature of the dyadic parent–child relationship. In line with this, parents often have the added sense of responsibility through their role as a parent, and as such can often experience complex feelings of guilt (De Young et al., 2014). Scheeringa and Zeanah (2001) proposed a bidirectional model of PTSD between parent and child, termed “relational PTSD”. This frames PTSD within the context of the attachment relationship which is considered fundamental to child development and general functioning (Groh et al., 2017). The relational model suggests that child trauma effects both the parent and the child, with their subsequent distress impacting one another. In addition, the model refers to the structural and systemic factors which may influence, and be influenced by, the interaction between parent and child PTSD. Parents experiencing PTSD are presented with additional challenges to maintain sensitively attuned parenting towards their children, given the debilitating impact of their own mental health. PTSD in adults has also been found to be associated with poor social support, socio-economic status, employment status and other structural and systemic factors (Brewin et al., 2000), which may also contribute to the co-occurrence of parental and child PTSD. Research suggests parents are more likely to display disconnected and insensitive parenting behaviours, which in turn impacts on child attachment security (van Ee et al., 2016).

### Prevalence and Risk Factors

In studies of post-traumatic stress symptoms in parents following their child’s single-incident trauma, prevalence rates have been reported to range greatly, from 0% (Fukunishi, 1998) to 52% (Landolt et al., 1998), and are often derived from different methods of assessment (e.g. clinical interview or self-report questionnaire). Furthermore, studies of risk factors for PTSD symptomology in parents have included multi-factorial assessments of pre-trauma factors, subjective and objective trauma characteristics, peri-traumatic factors and post-traumatic factors in relation to both the parent and child. Cognitive models of PTSD (Ehlers & Clark, 2000; Dalgleish, 2004) suggest subjective peri-traumatic experiences, such as perceived threat, play a significant role in the development of PTSD. This is supported by some studies of parental PTSD where factors such as parent perception of the trauma severity (Coakley et al., 2010), peri-traumatic dissociation (Hall et al., 2006), maladaptive cognitive appraisals and thought suppression (Hiller et al., 2016) are considered key. Other research suggests demographics associated with the parent (e.g. female gender; Balluffi et al., 2004) or the child (e.g. male gender; Martin-Herz et al., 2012) are important factors. Furthermore, some studies report factors associated with the trauma itself, such as severity (Rees et al., 2004), or with the post-trauma psychological reaction of the parent, such as depression (Kassam-Adams et al., 2015) and anxiety (Hall et al., 2006), or the child, such as PTSD (Landolt et al., 2003) and depression (Kassam-Adams et al., 2015), are key factors associated with parental PTSD. The literature indicates an array of possible correlates for parental PTSD; all of which suggest greatly varied effect sizes between studies, meaning the generalisability of single results may be questionable.

### Aims

The present review aimed to conduct a comprehensive search and collation of empirical research around parental PTSD following a child’s acute trauma. The review used a meta-analytic approach to estimate the *rates* of PTSD in parents following their child’s acute single-incident trauma, whilst also collating findings concerning *correlates* for developing PTSD in parents. The review also considers differences based on parental role (i.e. mothers and fathers), and the assessment method of PTSD to explore the impact this has on estimates. Developing a more reliable understanding of the current rates and risk correlates for PTSD in parents following their child’s trauma is of clinical importance, both for the parent and the child. Understanding the factors which may increase a parent’s risk of developing PTSD post-trauma could allow for better assessment, treatment and intervention for families, reducing the adverse outcomes for parents and children following traumatic events. The review will also have theoretical implications, providing a more cognisant account of the current understanding of parental PTSD, with suggestions for future research where necessary.

## Method

Prior to commencing the formal review searches, the protocol for this review was pre-registered on PROSPERO (Reference: CRD42018099578). The findings presented here are solely focussed on parents’ post-traumatic reactions to acute and/or single-incident trauma; findings related to trauma within the context of a child’s long-term health condition will be reported separately.

### Search Strategy

Articles in English language, published in peer-review journals between 1980 (when PTSD was first defined as a diagnosis in the Diagnostic and Statistical Manual of Mental Disorders, 3rd Edition (DSM-III); American Psychiatric Association, 1980) and June 2018 were considered for inclusion. Relevant studies were identified through a systematic search of leading psychological and medical databases, including MEDLINE (EBSCO), PsycINFO and Published International Literature on Traumatic Stress (PILOTS). The following search terms were used: (Parent* OR carer* OR caregiver* OR “care giver” OR mother* OR father* OR Maternal* OR Paternal*) AND (Child* OR “young person*” OR adoles* OR teen* OR infant* OR toddler* OR “young adult” OR “school child*” OR kid* OR juvenile* OR youth* OR pre-school*) AND (PTSD OR post-trauma* OR post trauma* OR posttrauma* OR trauma* OR “traumatic stress” OR Depress* OR “mood disorder*”) AND (Trauma* OR neglect* OR maltreat* OR abuse OR illness OR Disaster* OR violen* OR accident* OR war* OR assault* OR injur*).

All search terms were run by ‘Abstract and Title’ and Medical Subject Headings (MeSH Terms) were used for each individual search word. MeSH terms work similarly to a thesaurus to enhance the exploration of the vocabulary used within the searching to ensure a thorough, rigorous search strategy. See Supplementary Material 1 for reference list of papers included in the analysis, but not referenced in the text.

### Inclusion and Exclusion Criteria

To be considered for inclusion in the review, studies had to present data on the rate and/or identified correlates for parental PTSD, following their child’s trauma. The prevalence rate was operationalised as the number of participants who scored above clinical cut off on a validated measure of PTSD, or who met diagnostic criteria for PTSD through clinical interview. Correlates were defined as any reported variable associated with PTSD symptoms (i.e. correlational data) or used to compare PTSD symptoms in two groups. Trauma for this review was defined as a single-incident trauma, not considered as part of a pre-existing condition, for example, accidental injury or road traffic collision. Whilst it is recognised many children in the included samples may have experienced multiple traumatic incidents within the context of their admission to hospital for example, this study is specifically focussing on the admission, or incident as a ‘traumatic event’. The purpose for this is not to exclude those children who may have experienced more complex trauma (for example abuse or neglect) but to emphasise the uniqueness of these experiences within the parent–child relationship; therefore, we feel these would benefit from further independent review. The age range for children within the samples was set at 0–18 years. Articles were excluded from the review for any of the following reasons:

- The studies measured acute responses to trauma within the first month post trauma, rather than PTSD, which can only be diagnosed after 1 month (in line with DSM-5 criteria for PTSD).
- The study presented data related to parents’ PTSD symptoms which were not specifically related to their child’s trauma (e.g. from their own trauma history).
- Due to the complicating factors of grief in assessing PTSD in parents (Nakajima et al., 2012), studies in which the child died before PTSD was assessed were excluded.
- Although studies where the focus is around new-born children (e.g. trauma associated with neonatal intensive care) were included, those which focussed purely on birth trauma were excluded as birth was considered the adult’s trauma.
- If the sample included a parent who was the perpetrator of the traumatic incident, due to the complicating factor of this, perhaps representing developmental or relational trauma, or the children being removed from their parents’ care, thus not representing single-incident trauma.
- If the study reported insufficient data to calculate the prevalence rate or effect sizes.
- Where the aim of the study was to investigate the efficacy of treatment (e.g. randomised controlled trial) or where the sample selected participants for the presence of PTSD.
- Review articles, single case studies, dissertations, books, or other systematic reviews.
- Solely reviewed past research or purely qualitative methodology.
- Studies where the child’s trauma was associated with a medical/long-term condition (e.g. diagnosis of cancer) will be considered in a separate report. Some studies are defined as having a ‘mixed trauma’ sample; these are defined as those individuals who may have experienced a single traumatic event, however, the sample may also have included multiple traumatic events or where the child may have been diagnosed with a long-term condition, where this has not been directly specified in the paper. These samples were included when over 50% of the sample had reportedly experienced a single-incident trauma and are also reported within another review specifically looking at parental PTSD within the context of their child’s long-term health condition.

### Data Extraction

All papers were screened, and data were extracted by two independent researchers. Any queries were discussed and resolved through joint agreement. On the few occasions where further disagreement or uncertainty was evident, a third researcher (R.M-S) was involved in the final decision.

A data extraction database was used to record the following items of interest for inclusion in the meta-analysis: (a) article details (i.e. author, publication year, title, journal), (b) study design setting and recruitment method, (c) sample description (including number eligible to take part, sample size), (d) demographic information, (e) type and detail of index trauma experienced, (f) time since trauma to PTSD assessment and follow-up, (g) details of PTSD assessment method, (h) prevalence rate data (if reported) and (i) predictor/risk factor/correlate result statistics reported (effect sizes if provided or alternative statistics necessary to compute effect sizes). On extracting the data, a number of rules were adhered to in order to manage any uncertainty in the extraction and coding process and ensure consistency. If longitudinal studies presented assessment data on parental PTSD at multiple time points, effect sizes were derived from the time point nearest to the traumatic event, as long as it was more than one month after the event and subsequent assessments were excluded.

Further detailed information on data extraction procedures are provided in Supplementary Material 2.

### Data Synthesis

When prevalence rate and PTSD correlate data were gathered using multiple methods, these were combined using statistical transformations to account for any potential bias or skew in the results (Borenstein, 2009). When studies reported a non-significant result in the text, but did not report an effect size, an effect size of 0 was assigned, in order to reduce the risk of reporting bias. Whilst this strategy is sometimes considered conservative, and thus may result in underestimations of the actual effect sizes (Durlak & Lipsey, 1991), this approach is also considered more inclusive and thus favourable to simply excluding non-significant results from the analysis as this would likely bias the result by overestimating effect sizes (Rosenthal, 1995).

### Data Coding

For the purpose of this review, Pearson’s correlation coefficient, ‘*r*’, was used as the effect size of interest. The majority of studies reported Pearson’s *r* coefficients. However, where these coefficients were not reported, every effort was made to ensure data reported were included to ensure a more representative sample of results. This included computing effect sizes from means and sample sizes, *t*, *d*, eta, odds ratios, chi-squared and standardised regression (*β*) coefficients (Cohen, 1988; Borstein et al., 2009; Rosenthal, 1994). Data were interpreted using the conventional approach in which a ‘small’ effect is approximately *r* = 0.1, medium effect is approximately *r* = 0.3 and a large effect is approximately *r* = 0.5 or higher (Cohen, 1988).

### Quality Assessment of Risk and Bias

In order to assess the quality and risk of bias in the final included studies, a tool was developed based on the Assessment Tool for Observational Cohort and Cross-Sectional Studies (National Heart Lung and Blood Institute, 2014), the Quality Appraisal Checklist for Studies Reporting Correlations and Associations (NICE, 2012) and reviewing tools used in other prevalence rate and risk factors studies (e.g. Hoy et al., 2012; Munn et al., 2014). The assessment framework consisted of 12 items addressing three areas of interest: population (e.g. how well this was described and participation rates); outcomes (e.g. whether measures of PTSD and possible correlates were valid and reliable); and analyses (e.g. were the correct statistical analyses used); a copy can be found in Supplementary Material 3. Each item was given a score of 0–2, with 0 indicating low quality, and thus high bias, and 2 indicating high quality and thus low bias. Scores were summed to provide an overall quality score for each paper. For the papers where a question did not apply (e.g. those that did not report prevalence rate data), the total scores were pro-rated to ensure consistency. Papers with scores of 0–8 were considered low quality (high risk of bias), scores of 9–16 were considered medium quality (moderate risk of bias) and scores of 17–24 were considered high quality (low risk of bias). The first author completed quality ratings for all studies and the third author acted as a second rater for a random selection of 15 studies (37%). Inter-rater reliability of the scale was assessed for agreement between the rater’s scores on each of the double-rated studies. Inter-rater reliability for the quality scores was calculated with 37% of studies (*n* = 15), which indicated 98.6% agreement on all items (Intraclass correlation = 0.98, 95% CI 0.96–0.99).

### Meta-analytic Method

The meta-analysis of prevalence rate estimates was carried out using OpenMeta [Analyst] software (Wallace et al., 2012), whereas the meta-analysis of correlates was conducted using interface software MAVIS (version 1.1.3) (Hamilton, 2017); both applications run the meta-analysis using ‘R’ (version 3.43) utilising the ‘metafor’ (version 2.0.0) package (Viechtbauer, 2010). Random effects models were used due to the presumed variance in effect sizes extracted from each study.

Estimates of both PTSD rates and correlates were arcsine transformed to prevent the confidence intervals of studies with low PTSD rate estimates falling below zero (Barendregt et al., 2013). A separate meta-analysis was run for each correlates and *r* was used as the effect size reported as this was considered the most easily interpretable.

Moderator and sensitivity analyses were used to explore whether study characteristics and risk of bias impacted the strength of the effect sizes found. Moderator analyses for the prevalence rate estimates were planned for assessment method of PTSD (interview vs questionnaire), trauma type and parent role (mothers vs fathers). For both PTSD rate and correlates estimates, sensitivity analyses were planned to assess the risk of bias and impact of mixed trauma samples on the results found. This included re-running the analyses whilst excluding studies with a high risk of bias, and again excluding those which were considered a mixed, or ambiguous, trauma sample (i.e. they potentially included parents whose children had been subjected to medical trauma). Meta-regression analyses were conducted to test for statistical significance in any differences found.

## Results

**Fig. 1:**
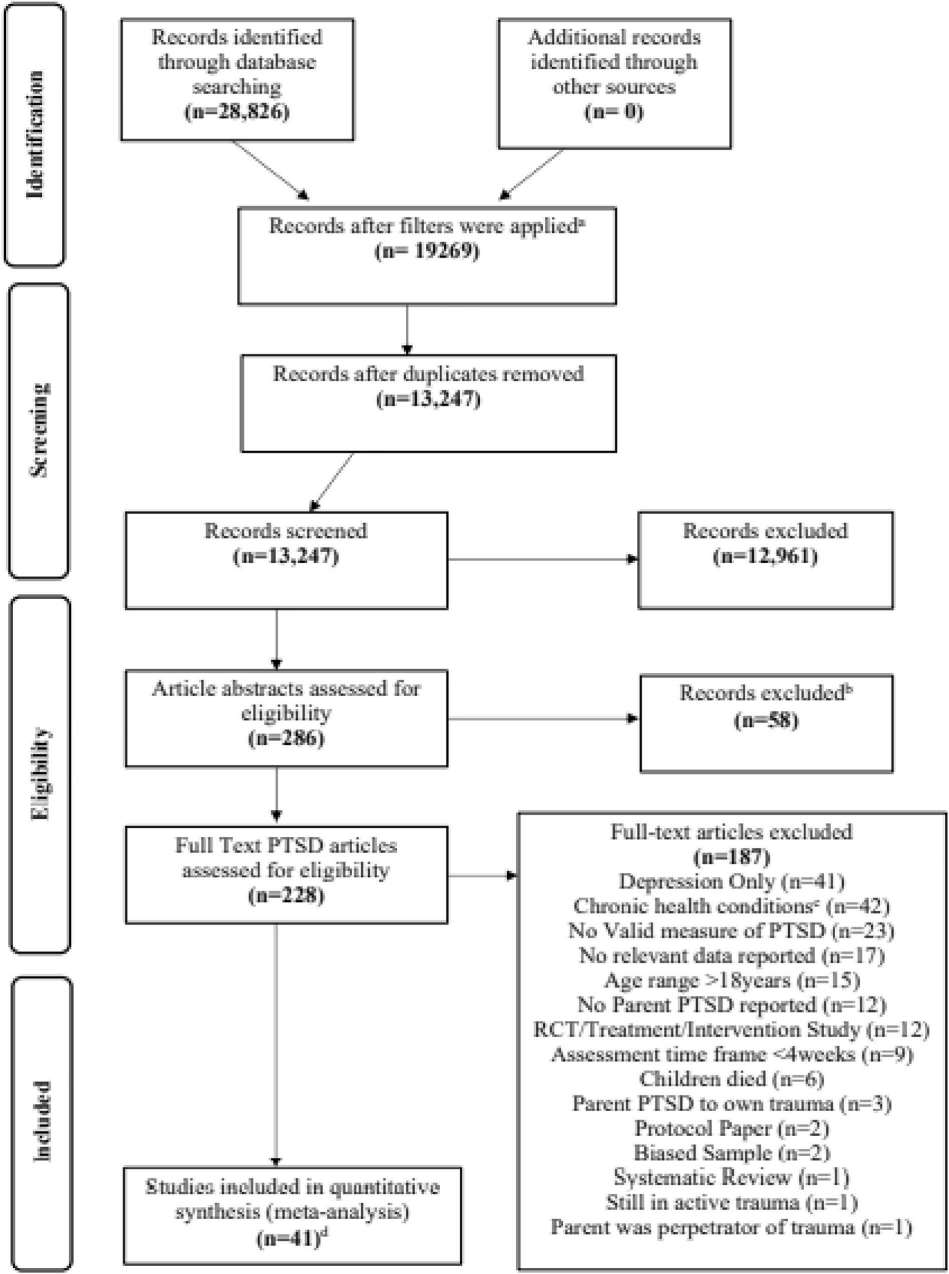
PRISMA diagram detailing the process of study selection. ^a^Filters applied included English Language, published in 1980 onwards; peer reviewed, Human studies only, exclude dissertations. ^b^Excluded as clearly did not meet study inclusion/exclusion criteria from the abstract. ^c^These papers used within another meta-analysis. ^d^Final studies include 4 papers merged with other papers due to replicated samples

Forty-one studies were included in the final quantitative synthesis; however, four articles were merged with others due to repeated samples, leaving total number of 37 samples included in the review. See the Preferred Reporting Items for Systematic Reviews and Meta-Analyses (PRISMA) diagram (Fig. 1) for the study selection, exclusion and inclusion process. Of these, 34 articles were included in the estimated PSTD prevalence rate analysis and 36 contributed to the associated analysis. Table 1 provides details of the design characteristics of each study included in the meta-analysis. 62.2% of studies had a longitudinal design, compared to 37.8% cross-sectional. 89.2% used self-report and 10.8% used interview. 13.5% of studies focussed on trauma type Road Traffic Accident (RTA), 21.6% on burns trauma type, 10.8% focussed on PICU admissions, 8.1% focussed on NICU admissions, 10.8% focussed on injury, 2.7% focussed on disasters, 5.4% focussed on Emergency Department admissions and 27.0% focussed on mixed trauma type. Sample size ranged from 460 to 16 participants, with the average sample size being 120.5. Timing of assessment ranged from 4 weeks to 7.32 years post trauma.

**Table 1:** Overview of study design included studies, methods of assessment, quality ratings and rates of PTSD in the parent samples

Study	Trauma type	Sample size	PTSD measure	Timing of PTSD assessment	Method of assessment	Study design	Location	PTSD rates		Risk of bias score	Risk of bias category
								*N*	%		
Allenou et al. (2010)	RTA	100	PCL-S	5 w	Self-report	Cross-sectional	France	14	14	16	Moderate
Bakker et al. (2013)	Burn	279	IES	3 m	Self-report	Longitudinal	Netherlands	59	21	18	Low
Balluffi et al. (2004)	PICU	161	PCL-S	Median = 4 m	Self-report	Longitudinal	USA	33	21	15	Moderate
Binder et al. (2011)	NICU	40	IES-R	1 m	Self-report	Longitudinal	USA	12	30	8	High
Bronner et al. (2008)	PICU	247	SRS-PTSD	3 m	Self-report	Longitudinal	Netherlands	31	13	19	Low
Bryant et al. (2004)	RTA	80	PDS	3 m	Self-report	Longitudinal	UK	2	3	18	Low
Chang et al. (2016)	NICU	102	IES-R	Mean = 21.5w	Self-report	Cross-Sectional	Taiwan & China	26	26	14	Moderate
Coakley et al. (2010)*	Mixed	51	PCL	4 w	Self-report	Cross-Sectional	USA	NR		17	Low
De Vries et al. (1999)	RTA	102	PCL	7–12 m	Self-report	Cross-Sectional	USA	15	15	14	Moderate
De Young et al. (2014)	Burn	120	PDS	1 m	Self-report	Longitudinal	Australia	25	21	11	Moderate
Egberts et al. (2016/2016)/Pan et al. (2015)	Burn	202	IES	3 m	Self-report	Longitudinal	Netherlands	36	22	16*	Moderate
Franck et al. (2015)	Mixed	107	IES-R	3 m	Self-report	Cross-Sectional	UK	23	22	18	Low
Fukunishi (1998)	Burn	16	SCID	4 years	Interview	Longitudinal	Japan	0	0	11**	Moderate
Hall et al. (2006)	Burn	62	PCL-C	3 m	Self-report	Longitudinal	USA	6	10	15	Moderate
Kassam-Adams et al. (2009)	Mixed	251	PCL	Mean = 6.5 m	Self-report	Longitudinal	USA	19	8	18	Low
Kassam-Adams et al. (2015)	Mixed	170	PCL	Mean = 5.3 m	Self-report	Cross-Sectional	USA	8	5	12	Moderate
Kubota et al. (2016)	NICU	72	IES-R	NR	Self-report	Cross-Sectional	Japan	14	19	13	Moderate
Landolt et al. (1998)	Mixed	29	PSS	6–8 w	Self-report	Cross-Sectional	Switzerland	15	52	15	Moderate
Landolt et al. (2003)	Mixed	355	PDS	5–6 w	Self-report	Cross-Sectional	Switzerland	71	20	16	Moderate
Landolt et al. (2012)	Mixed	460	PDS	5–6 w	Self-report	Longitudinal	Switzerland	111	24	17	Low
LeDoux et al. (1998)	Burn	35	IES	1–5 years	Self-report	Cross-Sectional	USA	4	11	5	High
Lefkowitz et al. (2010)	NICU	85	PCL	> 30 days	Self-report	Longitudinal	USA	11	13	14	Moderate
Martin-Herz et al. (2012)	Injury	92	PCL-C	2 m	Self-report	Longitudinal	USA	14	15	15	Moderate
Meiser-Stedman et al. (2017)/Hiller et al. (2016)	RTA	108/56	PDS	6 m	Self-report	Longitudinal	UK	13	11	17	Low
Mirzamani & Bolton (2002)	Disaster	37	PSS	3 m	Self-report	Longitudinal	Greece	13	35	12	Moderate
Nugent et al. (2007)	Injury	82	IES-R	6 w	Self-report	Longitudinal	USA	8	10	16	Moderate
Ostrowski et al. (2007)	ED	61	CAPS	6 w	Interview	Longitudinal	USA	1	2	17	Low
Ostrowski et al. (2011)*	ED	54	CAPS	6 w	Interview	Longitudinal	USA	NR		16	Moderate
Rees et al. (2004)	PICU	35	IES	6–12 m	Self-report	Cross-Sectional	UK	9	26	16	Moderate
Ribi et al. (2007)	Mixed	139	PDS	4–6 w	Self-report	Longitudinal	Switzerland	26	19	13	Moderate
Rizzone et al. (1994)	Burn	25	SCID	Mean = 7.32 years	Interview	Cross-Sectional	USA	4	16	6	High
Rodriguez-Rey & Alsonso-Tapia (2017)	PICU	143	DTS	6 m	Self-report	Cross-Sectional	Spain	33	23	13	Moderate
Scheeringa et al. (2015)*	Mixed	62	DTS	Mean = 11.2 m	Self-report	Longitudinal	USA	NR		15	Moderate
Sturms et al. (2005)	RTA	79	IES	3 m	Self-report	Longitudinal	Netherlands	22	44	13	Moderate
Van Meijel et al. (2015)	Injury	135	IES-R	3 m	Self-report	Longitudinal	Netherlands	13	10	20**	Low
Willebrand & Sveen (2016/2016)	Burn	106	IES-R	4 years	Self-report	Cross-Sectional	Sweden	21	20	13*	Moderate
Winston et al. (2003)	Injury	162	PCL	6.5 m	Self-report	Longitudinal	America	25	15	12	Moderate

*TA* road traffic accident, *PICU* paediatric intensive care, *NICU* neonatal intensive care, *ED* emergency department, *Mixed* sample may include single-incident and/or chronic trauma, *PCL-S* post-traumatic stress disorder checklist specific, *IES* impact of events scale, *SRS-PTSD* self-rating scale for post-traumatic stress disorder, *PDS* post-traumatic diagnostic scale, *PCL* post-traumatic stress disorder checklist, *IES-R* impact of events scale-revised, *SCID* structured clinical interview for DSM, *PSS* post-traumatic stress disorder symptom scale, *CAPS* clinician administered post-traumatic stress disorder scale, *DTS* Davidson trauma scale, *NR* not reported

*Aggregated quality score, due to merged papers

**Pro-rata scores due to some quality questions not being applicable

Table 2 provides an overview of parent and child participant characteristics in the included studies, at time of data collection details. 81.1% of studies included both mothers and fathers, compared to 16.2% just mothers, and 2.7% just fathers. Parent’s age ranged from 29 to 48.6 years, however, many ages were not reported. The average number of children included were 103.7 with an age range of 24.6 weeks to 18 years. Limited information on race and ethnicity of parents, or their socio-economic status was available.

**Table 2:** Overview of parent and child participant characteristics in the included studies, at time of data collection

Study	No. (%) parents		Mean age of parents (years)		Parent race (black and minority ethnic)	Parent low socio-economic status*	No of children	Age of children	
	Mothers	Fathers	Mothers	Fathers	*N* (%)	*N* (%)		Mean (SD)	Range
Allenou et al. (2010)	72 (72.0)	28 (28.0)	41.7	40.9	6 (8.3)	17 (23.6)	72	12.4 years (2.6)	8–17 years
Bakker et al. (2013)	186 (53.9)	159 (46.1)	31.9	35.7	NR	NR	198	1.8 years (0.9)	0.7–4.6 years
Balluffi et al. (2004)	132 (82.0)	29 (18.0)	NR	NR	67 (24.6)	NR	NR	NR	0–17 years
Binder et al. (2011)	20 (50.0)	20 (50.0)	35.0	NR	NR	NR	40	29.4 weeks (NR)	24.6–34 weeks
Bronner et al. (2008)	140 (56.7)	107 (43.3)	NR	NR	NR	NR	144	1.07 years (NR)	NR
Bryant et al. (2004)	80 (98.7)	1 (1.3)	NR	NR	NR	26 (32.1)	86	12.3 years (2.9)	5–16 years
Chang et al. (2016)	100 (100.0)	0 (0.0)	34.3	NR	NR	Education Level = 7 (6.7) Unemployed = 47 (46.1) Low Income = 52 (51.0)	102	NR	NR
Coakley et al. (2010)*	16 (31.3)	35 (68.6)	NR	NR	6 (11.8)	14 (27.5)	51	NR	8–15 years
De Vries et al. (1999)	102 parents*		NR	NR	NR	NR	102	9.4 years (3.5)	3–17 years
De Young et al. (2014)	111 (92.5)	9 (7.5)	32.9	NR	NR	50 (41.7)	120	2.7 years (1.49)	1–6 years
Egberts et al. (2016/2016)/ Pan et al. (2015)	114 (56.4)	88 (43.6)	NR	NR	NR	36 (17.8)	103	14 years (2.0)	9.5–17.8 years
Franck et al. (2015)	91 (85.1)	16 (14.9)	NR	NR	12 (11.2)	23 (51)	NR	8.3 years (6.1)	0–18 years
Fukunishi (1998)	16 (100.0)	0 (0.0)	37.5	NR	NR	NR	16	8.2 years (3.0)	NR
Hall et al. (2006)	54 (87.1)	8 (12.9)	NR	NR	21 (33.9)	NR	NR	1.5 years (NR)	6–17 years
Kassam-Adams et al. (2009)	226 (90.0)	25 (10.0)	NR	NR	201 (60.2)	NR	251	9.7 years (3.2)	5–17 years
Kassam-Adams et al. (2015)	132 (74.2)	46 (25.8)	NR	NR	NR	NR	178	11.5 years (2.6)	8–17 years
Kubota et al. (2016)	72 (100.0)	0 (0.00)	NR	NR	NR	NR	72	NR	6–17 years
Landolt et al. (1998)	29 parents*		NR	NR	NR	NR	34	10.7 years (3.2)**	5–16 years
Landolt et al. (2003)	180 (50.7)	175 (49.3)	NR	NR	NR	NR	209	10.0 years (2.3)	6.5–14.5 years
Landolt et al. (2012)	239 (52.0)	221 (48.0)	NR	NR	NR	NR	287	10.36 years (2.5)	NR
LeDoux et al. (1998)	32 (91.4)	3 (8.6)	NR	NR	NR	NR	35	13.25 years (2.7)	9–18 years
Lefkowitz et al. (2010)	60 (70.6)	25 (29.4)	29	33	22 (25.9)	65 (76.5)	NR	NR	NR
Martin-Herz et al. (2012)	72 (78.3)	20 (21.7)	43.8*		17 (18.5)	NR	92	15.7 years (1.9)	12–18 years
Meiser-Stedman et al. (2017)/Hiller et al. (2016)	56 (82.1)	46 (17.9)	NR	NR	NR	NR	56	6.8 years (2.8)	2–10 years
Mirzamani & Bolton (2002)	37 (100.0)	0 (0.0)	48.6*		NR	NR	37	NR	NR
Nugent et al. (2007)	78 (95.1)	4 (4.9)	NR	NR	NR	NR	82	13.2 years (2.9)	8.0–17.9 years
Ostrowski et al. (2007)	61 (100.0)	0 (0.0)	NR	NR	NR	NR	61	13.3 years (3.0)**	NR
Ostrowski et al. (2011)*	99 parents*		NR	NR	NR	NR	118	12.2 years (3.0)	8–18 years
Rees et al. (2004)	68 parents*		NR	NR	NR	NR	68	PICU = 8.8 (7.1, 10.8) Non-PICU = 9.6 (8.0, 115)***	5–18 years
Ribi et al. (2007)	0 (0.0)	139 (100.0)	NR	NR	NR	13 (9.4)	139	10.0 years (2.4)	NR
Rizzone et al. (1994)	24 (96.0)	1 (4.0)	37.0*		3 (12)	NR	30	NR	NR
Rodriguez-Rey & Alsonso-Tapia (2017)	92 (64.3)	51 (35.7)	38.2*		NR	NR	99	59.6 months (61.8)	NR
Scheeringa et al. (2015)*	62 (100.0)	0 (0.0)	30.3	NR	NR	32 (52)	62	4.1 years (1.4)	NR
Sturms et al. (2005)	64 parents*		NR	NR	NR	NR	64	12.2 years (NR)	8–15 years
Van Meijel et al. (2015)	120 (76.9)	36 (23.1)	NR	NR	NR	NR	161	13.9 years (2.8)	8–17 years
Willebrand & Sveen (2016/2016)	79 (73.8)	28 (26.2)	NR	NR	NR	13 (12)	107	NR	NR
Winston et al. (2003)	162 parents*		NR	NR	NR	NR	147	11.4 years (2.6)	8–17 years

*NR* not reported

Low socio-economic status—captured by employment status, education level, family income dependent on the study

^*^Data for parents reported as individual data were not provided for mothers/fathers separately

^**^Data reported are combined scores from two groups (e.g. boys and girls, high risk and low risk) as presented in the original paper

^***^These data reported are the median (quartiles) for the two groups as mean (sd) were not reported

### Risk of Bias Assessment

**Fig. 2:**
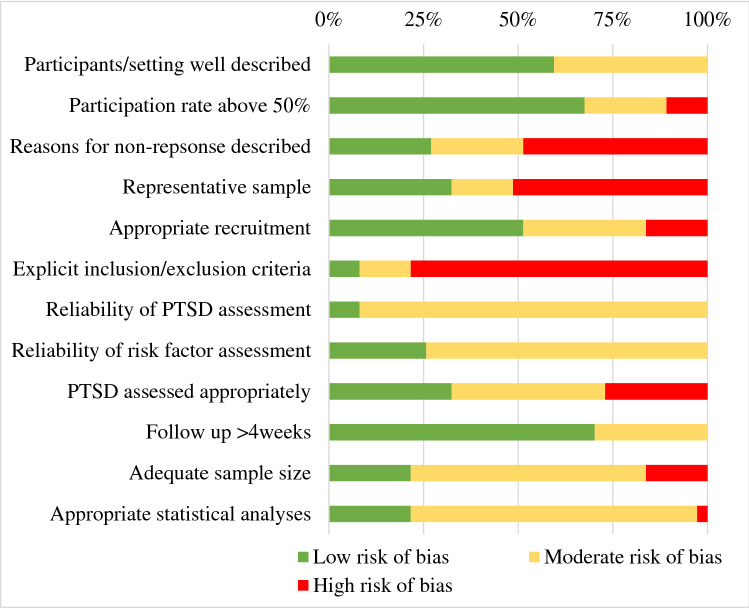
The proportion of studies rated low, moderate or high risk of bias on each of the quality assessment items

The overall risk of bias scores and category for each individual study can be seen in Table 1. Three studies were deemed to have high risk of bias, and thus low quality, 24 moderate risk of bias and ten were considered low risk of bias, and thus high quality. Figure 2. displays the proportion of studies rated low, moderate or high risk of bias for each of the individual quality assessment items.

### PTSD Prevalence Rates

With all 34 studies included in the PTSD prevalence rates analysis (*n* = 4158), the pooled estimates of PTSD rates in parents of children who have experienced a single-incident trauma was 17.0% (95% CI 14.1–20.0%) with considerable heterogeneity found between studies [*Q*(33) = 202.62, *p* < 0.001, *I*^2^ =83.7%]. Details of prevalence rates for each study can be found in Table 1.

### Subgroup and Moderator Analyses

**Fig. 3:**
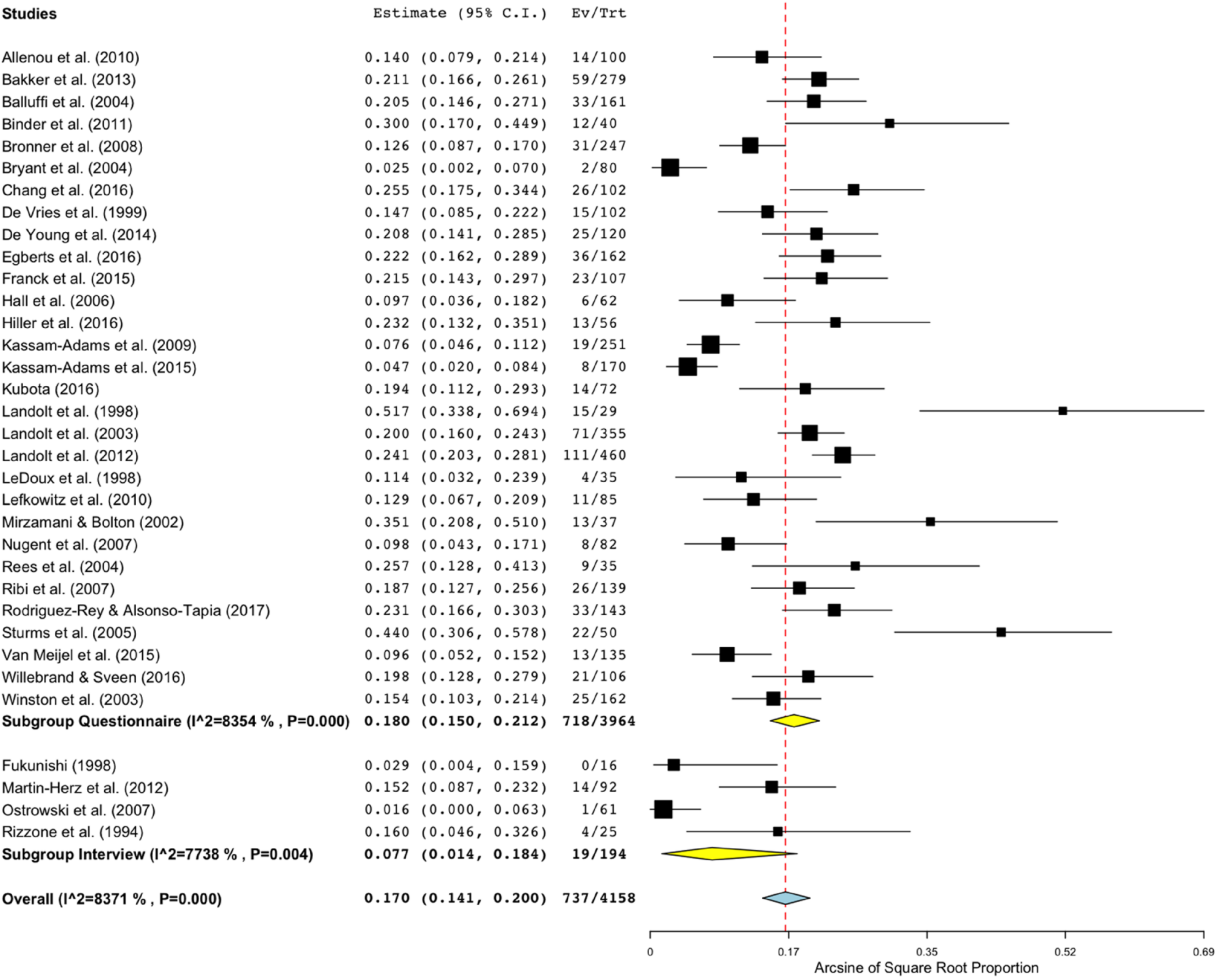
PTSD prevalence rate estimates for parents following their child’s trauma grouped by PTSD assessment method

Analyses of the PTSD rates estimates grouped by the method of PTSD assessment were conducted. A total of 30 studies assessed parent PTSD using a variety of self-report questionnaires, when considering these alone, the estimated PTSD rate was 18.0% (95% CI 15.0–21.2%) with considerable levels of heterogeneity [*Q*(29) = 176.18, *p* < 0.001, *I*^2^ =85.5%). For the remaining four studies that assessed parent PTSD using an interview format, the estimated PTSD rate was 7.7% (95% CI 1.4–18.4%), with considerable heterogeneity [*Q*(3) = 13.27, *p *= 0.004, *I*^2^ =77.4%]. See Fig. 3 for forest plot of total and assessment method subgroup prevalence rate estimates. Meta-regression analyses found that estimates of PTSD in parents were significantly higher when assessed by self-report questionnaire than by interview [*b* = −0.16, (95% CI −0.30, −0.02), *p* = 0.03].

Further subgroup and moderator analyses were conducted to explore any differences in PTSD rates based on trauma type and parent role; see Table 3 for estimates. With reference to trauma type, PTSD estimates appeared highest in parents of children who had been admitted to a Neonatal Intensive Care Unit (NICU). The lowest rates of PTSD were noted in those parents whose child had experienced an injury. Those who were reported in the mixed category, and thus may have exposure to more chronic trauma, or a long-term health condition were not notably different to other trauma type subgroups. It was also noteworthy that mothers appear to experience higher prevalence rates than fathers, however, these were not significant differences, and differences in sample size between mothers and fathers may influence these data.

**Table 3:** Prevalence estimates of PTSD in parents following their child’s trauma grouped by trauma type

Subgroup	*k*	Prevalence rate %	95% CI		SE	*p*	*z*	Q	df	*p*	*I*^*2*^
			LL	UL							
Trauma type											
RTA	5	17.3	0.07	0.31	0.08	0.001	5.13	42.27	4	< 0.001	90.54
Burn	8	17.5	0.14	0.22	0.03	< 0.001	15.74	13.64	7	0.058	48.69
PICU	4	19.2	0.13	0.26	0.04	< 0.001	11.26	9.84	3	0.020	69.50
NICU	4	21.1	0.14	0.29	0.05	< 0.001	10.61	6.94	3	0.074	56.75
Injury	4	12.6	0.10	0.16	0.03	< 0.001	14.49	3.49	3	0.322	14.02
Mixed	6	18.0	0.10	0.28	0.06	< 0.001	7.56	87.75	5	< 0.001	94.03
Other	3	16.1	0.02	0.40	0.14	0.003	2.95	29.01	2	< 0.001	93.10
Parent role											
Mother	14	20.1	0.15	0.26	0.04	< 0.001	12.49	94.70	13	< 0.001	86.27
Father	9	13.7	0.10	0.17	0.03	< 0.001	14.70	16.64	8	0.034	51.93

*RTA* road traffic accident, *PICU* paediatric intensive care unit, *NICU* neonatal intensive care unit

### Sensitivity Analyses

Sensitivity analyses were undertaken to consider the impact of risk of bias on the PTSD rate estimates. When removing the three studies with high risk of bias (Binderet al., 2011; Le Doux et al., 1998; Rizzone et al., 1994), the estimated incidence of parental PTSD was not dissimilar (16.8%, CI 13.9–20.0%); heterogeneity remained significant PTSD rate estimate results.

Further sensitivity analysis was conducted to explore the impact of studies in which the trauma type in the sample was mixed (e.g. Landolt et al., 2012), or where the trauma may not exclusively be considered as a single incident, for example, NICU/PICU sample. When these studies were excluded the PTSD estimate reduced to 14.4% (95% CI 10.8–18.5%); heterogeneity remained significant [*Q* (21) = 138.68, *p* < 0.001, *I*^2^ =84.9%]. Meta-regression analyses indicated the difference in PTSD estimate between exclusively acute/single-incident trauma sample and mixed samples were significantly different (*b* = − 0.071, (95% CI 0.012, 0.129), *p* = 0.018).

### Publication Bias

Publication bias was assessed via inspection of a funnel plot (see Supplementary Material 4). Observations suggest that the distribution of papers is asymmetrical, however, negative prevalence rates would be needed to get a symmetrical distribution. It seems that studies with larger samples tend to have smaller estimates, and larger estimates come from those with smaller samples. This may be because the studies are less representative of the wider population and are thus likely to produce less reliable and more bias results. This may suggest the asymmetry in the funnel plot represents a small sample bias, rather than a publication bias (Cuijpers, 2016).

### Correlates

Exploration of the 35 samples included in the correlate analysis generated a total of 194 effect sizes which were grouped to identify 32 correlates that were explored by two or more studies. The pooled sample size was 3874, with individual studies ranging from 25 to 355. Supplementary Material 5 provides an overview of the data extracted from each study for each correlate.

The main results of the estimates for each correlate can be seen in Table 4. These are grouped into objective trauma factors, factors relating to the parent, factors relating to the child and factors relating to the family.

**Table 4:** Individual meta-analyses of individual correlates for parent PTSD

Correlate	*k*	*n*	*r*	95% CI’s		*z*	*p*	*Q*	df	*p*	*I*^2^
				LL	UL						
Objective trauma factors											
Trauma severity	18	1976	0.10	0.02	0.18	2.50	0.0125	49.24	17	< 0.001	65.5
Hospital admission	3	359	0.10	− 0.08	0.28	1.09	0.2756	5.81	2	0.0548	65.6
Length of hospital admission	9	1252	0.16	0.03	0.28	2.49	0.0129	36.84	8	< 0.001	78.3
Parent direct exposure to trauma	7	748	0.17	− 0.02	0.35	1.78	0.0749	36.95	6	< 0.001	83.8
Parent factors											
Parent pre-trauma characteristics											
Older age	3	279	0.05	− 0.07	0.17	0.84	0.40	0.28	2	0.87	0.0
Female gender	8	1536	0.15	0.02	0.28	2.19	0.0287	43.82	7	< 0.001	84.0
Race (black and minority ethnic)	6	747	0.19	0.02	0.35	2.16	0.03	26.25	5	< 0.001	80.9
Low socio-economic status	5	691	− 0.05	− 0.18	0.09	− 0.68	0.5	11.91	4	0.02	66.4
Previous trauma or mental health difficulty	7	1061	0.23	0.09	0.36	3.21	0.001	28.80	6	< 0.001	79.2
Parent peri-trauma variables											
Perceived severity of trauma	7	807	0.29	0.16	0.40	4.43	< 0.001	18.52	6	0.005	67.6
Peritraumatic dissociation	3	218	0.23	0.03	0.41	2.24	0.0252	4.27	2	0.118	53.2
Acute stress disorder	5	791	0.49	0.32	0.63	5.13	< 0.001	32.43	4	< 0.001	87.7
Depression	7	769	0.59	0.38	0.74	4.79	< 0.001	88.08	6	< 0.001	93.2
Anxiety	4	368	0.45	0.17	0.66	3.01	0.0026	25.63	3	< 0.001	88.3
Stress	4	289	0.35	0.12	0.54	2.92	0.0035	10.56	3	0.0144	71.6
Psychological distress	5	413	0.29	− 0.02	0.55	1.82	0.0687	41.05	4	< 0.001	90.3
Negative coping style	2	246	0.43	0.78	0.57	5.05	< 0.001	1.99	1	0.1581	49.8
Avoidance	2	162	0.27	0.07	0.45	2.60	0.0094	1.61	1	0.2046	37.9
Alcohol use	2	199	0.09	− 0.05	0.23	1.27	0.2036	0.46	1	0.4959	0.0
Sense of blame/guilt	2	176	0.16	− 0.10	0.41	1.20	0.2299	2.85	1	0.0913	64.9
Neuroticism	2	241	0.40	0.05	0.67	2.23	0.0257	8.04	1	0.0046	87.6
Child factors											
Child pre-trauma characteristics											
Younger age	13	1750	− 0.08	− 0.13	−0.02	− 2.49	0.0128	17.35	12	0.137	30.8
Male gender	13	1589	0.07	0.01	0.14	2.08	0.0375	21.19	12	0.0476	43.4
Previous trauma/hospital admission	7	800	0.17	0.08	0.25	3.82	< 0.001	8.45	6	0.2069	29.0
Child trauma-related variables											
Medical complications	6	750	0.23	0.14	0.32	5.04	< 0.001	7.37	5	0.1947	32.1
Child post-trauma variables											
Acute stress disorder	3	423	0.12	−0.09	0.31	1.11	0.2689	7.75	2	0.0207	74.2
Post-traumatic stress disorder	15	1707	0.36	0.22	0.46	5.08	< 0.001	108.64	14	< 0.001	87.1
Externalising problems	5	551	0.20	0.10	0.30	3.95	< 0.001	5.47	4	0.2422	26.9
Poorer recovery	6	1012	0.27	0.21	0.33	8.79	< 0.001	2.15	5	0.8287	0.0
Comorbid psychological problem	4	538	0.21	−0.01	0.42	1.83	0.0666	21.07	3	< 0.001	85.8
Family factors											
Poor family functioning	8	829	0.23	0.07	0.37	2.77	0.0057	36.76	7	< 0.001	81.0
Lack of social support	3	238	− 0.08	− 0.21	0.05	− 1.22	0.2241	1.23	2	0.54	0.0

*k* number of studies, *LL* lower limit, *UL* upper limit

### Objective Trauma Factors

All objective trauma factors yielded a small effect size estimate (i.e. < 0.3); with trauma severity and length of hospital admission demonstrating statistical significance.

### Parental Factors

Parent factors were groups into pre-trauma and peri-trauma variables. Parental previous trauma or mental health difficulty, female gender, and individuals of Black and Minority Ethnic (BME) race were identified as significant pre-trauma correlates for PTSD, all with small effect sizes. Parental peri-trauma factors yielded a range of effect size estimates with perceived severity of trauma, peri-traumatic dissociation, acute stress disorder, depression, anxiety, stress, negative coping style, avoidance and neuroticism demonstrating statistical significance. Parental psychological factors (indication of acute stress disorder, depression, and anxiety) yielded large effect sizes (i.e. > 0.5).

### Child Factors

Child-related variables were grouped into child pre-trauma factors, trauma-related variables, and child post-trauma variables. Child-related pre-trauma factors all displayed small effect size estimates, and all were found statistically significant correlates for parental PTSD. The only child trauma-related variable (medical complications) was found to yield a small and statistically significant effect size. Child post-trauma variables mostly yielded medium effect sizes (i.e. 0.3–0.5), with child PTSD, child externalising behaviour problems and child poorer recovery identified as statistically significant predictors.

### Family Factors

Lastly, family factors were explored in which poor family functioning was found to be statistically significant predictor of parental PTSD, with a small effect size estimate.

### Sensitivity Analysis

Due to the high heterogeneity between studies, further sensitivity analyses were conducted to consider the impact of risk of bias and mixed sample studies on the correlate estimates (see Supplementary Material 6 for results). Each correlate meta-analysis was rerun with studies rated high risk of bias excluded. The estimate for parent direct exposure to trauma increased and became statistically significant. The correlate for female parent gender was reduced and was no longer significant. The sensitivity analyses for high risk of bias did not change the significance of any other associated factors.

Sensitivity analyses were also performed removing the mixed sample studies which were also included in another meta-analysis around long-term conditions (see Supplementary Material 6 for results). This revealed a decrease in the correlates for length of hospital admission, female parent gender and parent anxiety, which were no longer statistically significant. The statistical significance of all other variables was not changed based on the sensitivity analysis. Four variables (parent stress, parent negative coping style, poor child recovery and lack of social support) could not be synthesised as there were too few studies.

## Discussion

This systematic review and meta-analysis summarised the currently available research pertaining to parental PTSD following their child’s single-incident acute trauma, exploring both PTSD prevalence rate estimates and correlates for parental PTSD development. The pooled samples of PTSD rate data, totalling 4158 participants, resulted in an estimate of 17.0% (95% CI 14.1–20.0%). However, estimates were found to be significantly higher when assessed through self-report questionnaires compared to clinical interview. There was no evidence of a significant difference in the rates of PTSD reported for mothers and fathers (20.1% and 13.7%, respectively), however, it was found that being a female parent was associated with the development of PTSD. Whilst prevalence rates do not differ, the data suggest female parents are more at risk of developing PTSD than male parents; this may reflect differences in statistical power for the moderator and correlate analysis. Only a subset of included studies reported prevalence estimates for mothers and fathers separately (14 for mothers and 8 for fathers) or were composed exclusively of one gender only. As many mothers are often the primary caregiver, it is important to note the differences in sample sizes which may influence these findings, and the high levels of heterogeneity across the samples; there were larger samples of mothers than father included in the analyses throughout. Only one study (Ribi et al., 2007) just focussed on fathers PTSD reactions, in comparison to all others where mothers were included in the sample. In addition, research suggests there are higher rates of PTSD in females more generally (Tolin & Foa, 2006; Brewin et al., 2000) which may also explain the difference in these findings. Lastly, the method of assessment of PTSD in the correlational analysis has included both continuous and categorical (or diagnostic ‘caseness’) measures which may in turn impact the findings. As such, future research to explore the difference and uniqueness of the mother/father roles and the impact on parental psychological functioning is recommended.

The sample size of the pooled studies for the assessment of correlates was large (3874 parents) which yielded a total of 194 effect sizes. Correlates of parental PTSD were grouped into 4 main areas: objective trauma factors, parent factors, child factors and those related to family. Parent- and child-related factors were also broken down into subgroups of pre-trauma, peri-trauma and post-trauma factors.

Objective trauma factors, those relating to the trauma itself (such as severity, exposure and hospital admission) had small effect sizes. This is consistent with the findings from other meta-analyses of risk factors in children (Trickey et al., 2012) and adults (Brewin et al., 2000; Ozer et al., 2003). Exploration of parent factors suggested that pre-trauma factors (such as individual demographics or age, gender, race and socio-economic status) had little effect on the development of parental PTSD, as did post-trauma alcohol use. Factors with small effect sizes included parental previous trauma or mental health difficulty, peri-trauma processing, such as perceived severity of the trauma, and peri-traumatic dissociation, and some post-trauma variables such as avoidance, sense of blame/guilt and psychological distress. Parent factors found to have a medium to large effect size were related to their post-trauma functioning, such as development of Acute Stress Disorder, Depression, Anxiety and Stress, having a negative coping style, or displaying neuroticism.

When considering factors related to the children included in the studies, similar patterns arose. Pre-trauma factors, such as child age, gender and previous admission to hospital, were found to have trivial or small effect sizes, alongside a post-trauma variable of the child’s development of Acute Stress Disorder. The experience of medical complications, child’s externalising problems, overall poorer recovery and comorbid psychological problems yielded small effect sizes, while child PTSD yielded a medium effect size. Lastly, factors related to the family as a whole showed that lack of social support had a trivial effect size, whereas poor family functioning resulted in a small effect on the development of PTSD in parents.

Factors related to the parent’s appraisal of, and psychological response to, their child’s trauma, and the child’s post-traumatic stress reaction and externalising behavioural response had larger effects. Of particular importance in understanding PTSD development in parents were larger effects found for the way parents appraised the severity of their child’s trauma, and the indication of psychological factors such as acute stress disorder, depression and anxiety.

The results provide support for the association between child and parent PTSD which is based on a relatively large number of studies. Interestingly, other child psychological factors (e.g. acute stress disorder and comorbid psychological problems) did not show large effects associated with the development of parental PTSD. Whilst this may relate to differences in the number of studies exploring these topics, it would be interesting for future research to further explore the complexity of PTSD across the parent–child relationship in comparison to other mental health presentations. In particular, research which explores the mechanisms through which this relationship operates would provide a greater understanding of the most effective way at targeting systemic interventions post-trauma. This is particularly noteworthy, given that family functioning was only found to yield a small protective effect. Perhaps the notion of family functioning fails to acknowledge or explore the uniqueness of the parent–child relationship and complexity of the parent role; further research is warranted to explore this in more depth.

### Clinical Implications and Suggestions for Future Research

The results from this review have some implications for both the theoretical and clinical understandings of PTSD in parents following their child’s single-incident acute trauma. Firstly, the results provide support for the dyadic relational impact of child trauma on both children and parents; supporting both Kazak et al. (2006) integrative model of paediatric traumatic stress and Scheeringa & Zeanah’s (2001) model of relational PTSD. Importantly, the findings suggest a relationship, and therefore the direction of this relationship is unclear, which may be bidirectional in nature. Despite this, the findings suggest that clinical services offering support to children following an acute trauma should not be solely focussed on the child, at the expense of the parents, given the relationship between child PTSD and parents psychological functioning. Alongside this, it is worth noting that some of the correlates explored showed little effect on parental and child PTSD, which may guide the process of targeted intervention. Similarly, to recommendations made by Scheeringa & Zeanah (2001), this review suggests the need for assessing and treating the family system as a whole, with an initial focus on supporting parent mental health alongside the child’s mental health. This is important as changes in the relationship between the parent and child may be fundamental to a change in child symptomology (Crockenberg & Leerkes, 2000), and change in parental symptomology is likely to contextually change their interaction and ability to attune to the needs of their child. As has been highlighted in Kazak’s (2006) model, trauma occurs to children in family systems, therefore we argue that assessment and treatment of child PTSD should occur within the context of these systems also. Further research into appropriate, and clinically accessible, ways of assessing indicators of adverse reactions in the early stages of post-trauma is recommended.

In many of the included studies, limited and inconsistent information regarding parental race/ethnicity and socio-economic status was reported, thus questioning the representativeness of the samples included. It is recommended that this is sensitively considered when applying to clinical practice. Of those who did report on the diversity of the parent samples, rates of parents from BME or low socio-economic backgrounds appear low, therefore it is recommended that future research actively works to include these groups of often underrepresented samples in their studies, and considers the limitations of low diversity of their samples in their analyses.

Similar to what has been found in other explorations of PTSD correlates, our results suggest that demographic, pre-trauma and objective trauma factors are not particularly useful screening markers for PTSD (Brewin et al., 2000; Cox et al., 2008; Trickey et al., 2012). Instead, the results point towards a systemically informed psychosocial account of PTSD development. Whilst cognitive and behavioural models of PTSD are already well established in other populations (Ehlers & Clarke, 2000; Dalgleish, 2004), this study notes how little psychological factors, aside from comorbid psychological diagnoses, have been addressed within the child, and parent populations. Post-trauma cognitive processing and parenting behaviour were only considered by a very small number of studies (e.g. Hiller et al., 2016; Meiser-Stedman et al., 2017). Therefore, we strongly encourage further exploration of cognitive and behavioural aspects of post-trauma processing, the relational nature of these processes and the impact this has on both parent and child psychological functioning.

The present study excluded studies which solely focussed on post-traumatic depression in parents. Given our finding of parent depression as a significant PTSD correlate, with a large effect size, future research should look to exploring this further, to investigate PTSD rate and correlates for post-traumatic depression in parents. Furthermore, whilst this study provides an up-to date amalgamation of the current research on parental PTSD following their child’s trauma, what is not known is the directionality of this effect; longitudinal research is needed to explore trajectory of child–parent PTSD relationship.

### Implications for PSTD Measurement

It is important to consider the implications for the measurement of PTSD in parents from the results of this study. Notably, the estimates of PTSD rates were found to be higher when collected via a self-report measure, in comparison to those which were clinician reported. This leads to questions about the clinical usefulness of both methods of measurement; perhaps clinicians are under-detecting parental PTSD and therefore self-report measures may be preferable for higher rates in detection. However, many of the measures used were not tailored to the uniqueness of parental PTSD, and therefore further exploration of the validated of various assessment methods would be useful.

In addition, those parents where the child’s trauma was considered ‘mixed’ and therefore may have been more chronic in nature, or within the context of a long-term health condition, showed higher effect sizes. This suggests implications for the measurement and consideration of a child’s experience of complex trauma and the impact of this on parental PTSD rates. As such, further exploration of this more complex, chronic and systemic trauma exposure is warranted.

### Limitations

This review has several limitations. Firstly, it is important to acknowledge the high heterogeneity across studies both for the estimates of PTSD rates and correlates, with both sensitivity and moderator analyses failing to decrease this. This is likely to be attributed to the various ways PTSD and correlate variables were measured, the variability of trauma types included, the broad age range of the children, and variability in time between the traumatic event and assessment of PTSD across studies. It is important this may reflect possible differences in the data collected from the included studies using cross-sectional and longitudinal designs. Of the included studies, over 60% were of a longitudinal design, and therefore collected data on PTSD presentation over multiple time points. Whilst the review only included data at the time point closest to the traumatic incident (over 1 month), the possible impact of study design was not reviewed as a possible mediator of findings and may have influence on the effect sizes reported. Given the variability in PTSD assessment timing, future research may wish to explore timing of the assessment as a potential correlate for PTSD diagnosis.

Secondly, it is important to be cautious when interpreting the pooled correlate data for child psychological factors as most of the variables from these studies comprised parent-report measures. Whilst this is often the only way to explore factors related to children (particularly young children), it is acknowledged this may bias the results of the child-related variables; a gold standard approach would be to collect child self-report data. Alongside this, many of the correlates included in the study were only assessed by a small number of studies, which means conclusions drawn about these are limited. As previously mentioned, this is likely to be associated with the immaturity of this area of research, and with a lack of routine assessment of possible risk factor variables across studies. However, Valentine et al. (2010) argue that meta-analyses, even with ‘small *n*’, are more informative than not synthesising the results.

Whilst adding significantly to the current understanding of parental PTSD, it is important to note that many of the correlate effect size estimates were based on a small number of studies. The results should therefore be considered with caution as only four out of the 32 variables examined were assessed by more than 10 studies. This is similar to other meta-analytic reviews of risk factors for PTSD in children (Trickey et al., 2012) who highlighted a lack in routine examination of the same variables in multiple studies. In contrast, meta-analytic reviews within adult populations show much more routine PTSD assessment (e.g. Brewin et al., 2000), therefore rather than suggesting the need to discredit results, it is likely to reflect the immaturity of the PTSD literature in children and parents and highlights a need for further exploration of this research area.

In addition, it is important also to note the variability in heterogeneity across the correlate estimates (range 0.0–93.2%) with the majority showing significant heterogeneity across effect sizes from individual studies. This suggests that there is an apparent need for further investigation of the presented factors which our present knowledge is limited. Given this, the results of the meta-analysis need to be considered within the wider context of variability of effect sizes both within and between the studies for each correlate, which limits the generalisability of the findings. This provides clear avenues for future research into the impact of child trauma on parents.

## Conclusion

This systematic review and meta-analyses provide evidence that parents of children who experience a single-incident trauma are at risk of developing PTSD, with a significant minority meeting threshold for this condition. It provides estimates for various factors which place parents at a high risk of developing PTSD, associated with the trauma itself, the parent and the child. Whilst a range of effects were found, the evidence highlights the pre-trauma factors, of the parent and the child have a small effect on the development of PTSD in parents. Peri-trauma factors, and post-trauma psychological processing of both the parent and child were more effective predictors of Parental PTSD development, and in which objective trauma variables and individual demographics play a less significant role.

Despite these useful findings, the research in this area is limited, and thus further research in this clinically and theoretically important field is necessary, with particular attention paid to the exploration of the relational uniqueness of the parental role and key psychological processes which may provide further insight to various elements of parental PTSD.

## Supplementary Information

Below is the link to the electronic supplementary material.

Supplementary file1 (DOCX 88 kb)
