# Supplementary material for: Post-traumatic Stress Disorder in Parents Following Their Child’s Single-Event Trauma: A Meta-Analysis of Prevalence Rates and Risk Factor Correlates
Source: Clin Child Fam Psychol Rev. 2021 Sep 23;24(4):725–43. doi: 10.1007/s10567-021-00367-z (PMC8541994; doi:10.1007/s10567-021-00367-z)
Supplement: Supplementary file 1 — Supplementary file1 (DOCX 88 kb) [file 10567_2021_367_MOESM1_ESM.docx]

**Supplementary Material**

**Post-Traumatic Stress Disorder in Parents Following Their Child’s single-event Trauma: A Meta-Analysis of Prevalence and Risk Factors**

**Clinical Child and Family Psychology Review**

Authors: *Lucy A. Wilcoxon^1^ Richard Meiser-Stedman ^1^ Aaron Burgess ^1^

^1^Department of Clinical Psychology and Psychological Therapies, Norwich Medical School, University of East Anglia, NR4 7TJ, UK

*Corresponding Author email: [l.wilcoxon@uea.ac.uk](mailto:l.wilcoxon@uea.ac.uk)

**Supplementary Material 1:** Reference list of papers included in the analysis, but not referenced in the text.

*Allenou, C., Olliac, B., Bourdet-Loube’re, S., Brunet, A., David, A, C., Claudet, I., Lecoules, N., Roulet, P., Bui, E., & Birmes, P. (2010). Symptoms of traumatic stress in mothers of children victims of a motor vehicle accident. *Depression and Anxiety 27,* 652-657.

*Bakker, A., Van der Heijden, P, G, M., Van Son, M, J, M., & Van Loey, N, E, E. (2013). Course of traumatic stress reactions in couples after a burn event to their young child. *Health Psychology 32*(10), 1076-1083. http://dx.doi.org/10.1037/a0033983

*Bronner, M, B., Knoester, H., Bos, A, P., Last, B, F., & Grootenhuis, M, A. (2008). Follow-up after paediatric intensive care treatment: parental posttraumtic stress. *Acta Paediatrica, 97*, 181-186. DOI:10.1111/j.1651-2227.2007.00600.x

*Bryant, B., Mayou, R., Wiggs, L., Ehlers, A., & Stories, G. (2004). Psychological consequences of road traffic accidents for children and their mothers. *Psychological Medicine, 34*, 335-346. DOI: 10.1017/S0033291703001053

*Chang, H, P., Chen, J, Y., Huang, C, J., Huang, J, Y., Su, P, H., & Chen, V, C, H. (2016). Factors associated with post-traumatic symptoms in mothers of preterm infants. *Archives of Psychiatric Nursing, 30,* 96-101. http://dx.doi.org/10.1016/j.apnu.2015.08.019

*De Vries, A, P, J., Kassam-Adams, N., Cnaan, A., Sherman-Slate, E., Gallagher, P, R., & Winston, F, K. (1999). Looking beyond the physical injury: posttraumatic stress disorder in children and parents after pediatric traffic injury. *Pediatrics, 104*(6), 1293-1299. DOI: 10.1542/peds.104.6.1293

*Egberts, M, R., van de Schoot, R., Boekelaar, A., Hendrickx, H., Geenen, R., & Van Loey, N, E, E. (2016). Child and adolescent internalizing and externalizing problems 12 months postburn: the potential role of preburn functioning, parental posttraumatic stress, and informant bias. *European Journal of Child and Adolescent Psychiatry, 25*, 791-803. DOI 10.1007/s00787-015-0788-z

*Egberts, M, R., van de Schoot, R., Hendrickx, H., Geenen, R., & Van Loey, N, E, E. (2017). Parents’ posttraumatic stress after burns in their school-aged child: a prospective study. *Health Psychology 36*(5), 419-428. http://dx.doi.org/10.1037/hea0000448

*Franck, L, S., Wray, J., Gay, G., Dearmun, A, K., Lee, K., & Cooper, B, A. (2015). Predictors of parent post-traumatic stress symptoms after child hospitalisation on general pediatric wards: a prospective cohort study. *International Journal of Nursing Studies, 52,* 10-21. http://dx.doi.org/10.1016/j.ijnurstu.2014.06.011

*Kassam-Adams, N., Fleisher, C, L., & Winston, F, K. (2009). Acute stress disorder and posttraumatic stress disorder in parents of injured children. *Journal of Traumatic Stress, 22*(4), 294-302. DOI: 10.1002/jts.20424

*Kubota, A., Yamakawa, S., Yamamoto, E., Kosugi, M., Hirano, S., Shiraishi, J., … Yamaue, H. (2016). Major neonatal surgery: psychosocial consequences of the patient and mothers. *Journal of Pediatric Surgery, 51*, 364-367. http://dx.doi.org/10.1016/j.jpedsurg.2015.09.017

*Lefkowitz, D, S., Baxt, C., & Evans, J, R. (2010). Prevalence and correlates of posttraumatic stress and postpartum depression in parents of infants in the neonatal intensive care unit (NICU). *Journal of Clinical Psychology in Medical Settings, 17*, 230-237. DOI 10.1007/s10880-010-9202-7

*Mirzamani, M., & Bolton, D. (2002). PTSD symptoms of mothers following occurrence of a disaster affecting their children. *Psychological Reports, 90*, 431-438.

*Nugent, N, R., Ostrowski, S., Christopher, N, C., & Delahanty, D, L. (2007). Parental posttraumatic stress symptoms as a moderator of child’s acute biological response and subsequent posttraumatic stress symptoms in pediatric injury patients. *Journal of Pediatric Psychology, 32*(3), 309-318. doi:10.1093/jpepsy/jsl005

*Ostrowski, S, A., Christopher, N, C., & Delahanty, D, L. (2007). Brief report: the impact of maternal posttraumatic stress disorder symptoms and child gender on risk for persistent posttraumatic stress disorder symptoms in child trauma victims. *Journal of Pediatric Psychology, 32*(3), 338-342. doi:10.1093/jpepsy/jsl003

*Ostrowski, S, A., Ciesla, J, A., Lee, T, J., Irish, L., Christopher, N, C., & Delahanty, D, L. (2011). The impact of caregiver distress on the longitudinal development of child acute posttraumatic stress disorder symptoms in pediatric injury victims. *Journal of Pediatric Psychology, 36* (7), 806-815. doi:10.1093/jpepsy/jsq113

*Pan, R., Egberts, M, R., Castanheira Nascimento, L., Aparecida Rossi, L., Vandermeulen, E., Geenen, R., & Van Loey, N, E. (2015). Health-related quality of life in adolescent survivors of burns: agreement on self-reported and mother’ ad fathers’ perspectives. *Burns, 41*, 1107-1113. http://dx.doi.org/10.1016/j.burns.2014.12.011

*Rodriguez-Rey, R., & Alonso-Tapia, J. (2017). Relation between parental psychopathology and posttraumatic growth after a child’s admission to intensive car: two faces of the same coin? *Intensive and Critical Care Nursing, 43*, 156-161. http://dx.doi.org/10.1016/j.iccn.2017.08.005

*Scheeringa, M, S., Myers, L., Putnam, F, W., & Zeanah, C, H. (2015). Maternal factors as moderators or mediators of PTSD symptoms in very young children: a two-year prospective study. *Journal of Family Violence, 30*, 633-642.

*Sturms, L, M., van der Sluis, C, K., Stewart, R, E., Groothoff, J, W., Jan ten Duis, H., & Eisma, W, H. (2005). A prospective study on paediatric traffic injuries: health-related quality of life and post-traumatic stress. *Clinical Rehabilitation, 19*, 312-322. DOI: 10.1 191/026921 5505cr867oa

*Van Meijel, E, P, M., Gigengack, M, R., Verlinden, E., Opmeer, B, C., Heij, H, A., Goslings, J, C., … Lindauer, R, J, L. (2015). Predicting posttraumatic stress disorder in children and parents following accidental child injury: evaluation of the screening tool for early predictors of posttraumatic stress disorder (STEPP). *BMC Psychiatry, 15* (113). DOI 10.1186/s12888-015-0492-z

*Willebrand, M., & Sveen, J. (2016). Injury-related fear-avoidance and symptoms of posttraumatic stress in parents of children with burns. *Burns, 42*, 414-420. http://dx.doi.org/10.1016/j.burns.2015.08.004

*Willebrand, M., & Sveen, J. (2016). Perceived support in parents of children with burns. *General Hospital Psychiatry, 38*, 105-108. http://dx.doi.org/10.1016/j.genhosppsych.2015.10.004

*Winston, F, K., Kassam-Adams, N., Garcia-Espana, F., Ittenbach, R., & Cnaan, A. (2003). Screening for risk of persistent posttraumatic stress in injured children and their parents. *Journal of the American Medical Association, 290*(5), 643-649.

**Supplementary Material 2:** Detailed information on data extraction procedures.

On extracting the data, a number of rules were adhered to in order to manage any uncertainty in the extraction and coding process and ensure consistency. If longitudinal studies presented assessment data on parental PTSD at multiple time points, effect sizes were derived from the time point nearest to the traumatic event, as long as it was more than one month after the event and subsequent assessments were excluded. Data was only extracted for current, not past PTSD. For the extraction of prevalence estimate data, when articles either used two measures of PTSD (e.g. questionnaire and interview) or reported both categorical (diagnosis) and continuous (symptoms severity) measures, the categorical measures were prioritised due to their accuracy. However, when only continuous measures of PTSD were reported, prevalence estimates extracted included the ‘moderately severe’ and ‘severe’ categories. For the risk factor analysis, continuous measures of PTSD symptoms severity were prioritised due to their statistical advantages in predictive analyses. If studies had a mixed sample (including both acute/single incident trauma and long-term condition) efforts were made to extract the data just relating to parent PTSD following acute trauma. In one study (Ribi, Vollrath, Sennhauser, Gnehm, & Landolt, 2007) prevalence estimates for parental PTSD were reported separately for acute trauma and long-term injury, therefore just prevalence of the single trauma sample was extracted. Continuous measures of PTSD were prioritised for risk factor estimates but, in some studies, where continuous data was not presented categorical ‘case-ness’ was used.

In addition, data relating to risk factors for parental PTSD were only included if they were collected prior to or concurrently with the PTSD assessment. Different articles that reported results from the same data set were included in the review if the studies provided prevalence rates or effect size estimates for different risk-factors, this was to avoid any repetition resulting in biasing the sample. Data from the same sample was reported on four occasions, this is noted in the tables by merging those repeated samples together.

**Supplementary Material 3:** Copy of Quality Assessment Framework

Well addressed = 2

Partially addressed = 1

Poorly addressed/not addressed/not reported = 0

| **Assessed by:** | |
| --- | --- |
| **Section 1: Population** | |
| **1.1 Were participants and setting well described?** | |
| Information regarding the characteristics (age, gender, ethnicity) of the sample and trauma variables (type, severity, duration) are well described with the setting well reported (health setting, country, geography) | 2 |
| Some information regarding participants characteristics and trauma variables are reported, with limited information on the setting | 1 |
| Sample characteristics, trauma variables and setting information are not reported in any detail | 0 |
| **1.2 Was participation rate of those eligible at least 50%**? | |
| More than 50% of those eligible to participate took part | 2 |
| Less than 50% of those eligible to participate took part | 1 |
| The number of eligible potential participants was not reported | 0 |
| **1.3 Were reasons for non-response described?** | |
| Reasons for non-response were described with the number of those participants not responding reported | 2 |
| Reasons were described for non-responders but no numbers provided OR Numbers of non-responders are reported but with no reasons | 1 |
| Non-response rates were not reported in the study | 0 |
| **1.4 Was the sample representative – were there differences between those participants taking part and those not?** | |
| There were no significant differences in demographics or trauma variables between those participating and those not | 2 |
| Reported significant differences between those participating and those not | 1 |
| Differences between participants and those not taking part were not reported | 0 |
| **1.5 Were participants recruited in an appropriate way?** | |
| Consecutive or random sampling was used to recruit potential participants in person by the research team | 2 |
| Consecutive or random sampling was used to recruit potential participants via letter or phone call | 1 |
| Recruitment procedures were not reported in the study | 0 |
| **1.6 Were inclusion and exclusion criteria explicit and appropriate?** | |
| Inclusion and exclusion criteria were reported in detail with a clear rationale | 2 |
| Some information on inclusion and exclusion criteria were reported but lacked a rationale | 1 |
| Inclusion and exclusion criteria were not reported | 0 |
| **Section 2: Outcomes** | |
| **2.1 Was objective, standardised criteria used for the assessment of PTSD**? |  |
| A diagnostic interview was used which demonstrated good levels of reliability and validity in assessment of PTSD in parents, adhering to DSM-III, DSM-IV or DSM-5 criteria for PTSD | 2 |
| A self-report questionnaire used which demonstrated good levels of reliability and validity in the assessment of PTSD in parents, adhering to DSM-III, DSM-IV or DSM-5 criteria for PTSD | 1 |
| An observer-rated questionnaire/interview, self-report questionnaire without using DSM criteria, generic clinical interview was used, or measures used demonstrated poor reliability and/or validity | 0 |
| **2.2 Were risk factors assessed using reliable and valid measures** | |
| Risk factors for developing PTSD were assessed using a structured clinical interview or extracted from medical records (e.g. demographics, trauma related variables) or based on the physician/doctor/other professional | 2 |
| Risk factors for developing PTSD were assessed using reliable and validated self/parent-report outcome measures (including parent report of medical severity) | 1 |
| Risk factors were not based on reliable or valid measures | 0 |
| **2.3 Was PTSD (and risk factors) assessed appropriately (professional and setting)?** | |
| Assessment was carried out in person by an appropriately trained professional (e.g. clinical psychologist, psychiatrist, research nurse, trainee psychologist, psychological therapist, research assistant) at the most convenient location (e.g. participant’s home if discharged from hospital). Or if self-report measures were used, they were administered by a trained professional to participants or participants had the opportunity to ask questions or speak with a trained professional. | 2 |
| Assessment was carried out by a trained professional over the phone **AND/OR** child factors were assessed by proxy (e.g. parent). Or if self-report measures were used participants had the opportunity to speak with a trained/clinical professional over the phone. | 1 |
| Assessment was indirect (through other health care professionals) or participants had no opportunity to discuss self-report measures with a trained/clinical professional. OR information regarding location and person assessing PTSD and risk factors were not reported. | 0 |
| **2.4 Was follow-up time for PTSD assessment appropriate and meaningful?** | |
| An appropriate time frame (>4 weeks post trauma) was used when assessing for PTSD | 2 |
| PTSD assessment was undertaken >6 months post trauma | 1 |
| No information regarding time frame used when assessing PTSD was reported | 0 |
| **Section 3: Analyses** | |
| **3.1 Was the sample size adequate?** | |
| Sample size was adequate to detect prevalence and risk factors which was based on a sample size or power calculation (or based on consideration of previous studies) | 2 |
| Sample size was adequate without reference to sample size calculations or consideration of previous studies | 1 |
| Same size justification was not reported, or sample size was too small | 0 |
| **3.2 Was there appropriate statistical analysis used** | |
| Statistical methods used for analysis were appropriate, with confidence intervals at 95% reported for estimate | 2 |
| Statistical methods used for analysis were appropriate, but no confidence intervals were reported | 1 |
| Statistical methods used were inappropriate or the study lacked information on statistical methodology when reporting data | 0 |
| **Overall Risk of Bias** | /24 |

**Low risk of bias 17 -24**

**Moderate risk of bias 9 - 16**

**High risk of bias 0 - 8**

*This tool was developed based on previous quality checks and research (National Heart Lung and Blood Institute, 2014; NICE, 2012; Hoy et al., 2012; Munn, Moola, Riitana & Lisy, 2014).*

**Supplementary Material 4:** Funnel plot for Assessment of Publication Bias

**Supplementary Material 5**: An overview of the data extracted from each study for each correlate of parental PTSD

| **Correlate** | | | | **Article Name** | **Assessment of correlate** | ***k*** | **Mean *r*** | **N** | **Min.** | **Max.** |
| --- | --- | --- | --- | --- | --- | --- | --- | --- | --- | --- |
| Objective Trauma Factors | | | | | | | | | | |
|  |  | Trauma Severity | | | | | | | | |
|  |  |  | Balluffi et al. (2004) | | Paediatric Risk of Mortality Scale-III | 1 | 0.00 | 161 |  |  |
|  |  |  | Binder et al. (2011) | | Merge mother and father PTSD for Score for Neonatal Acute Physiology and Perinatal Risk Inventory | 4 | 0.15 | 40 | -0.001 | 0.32 |
|  |  |  | Bronner et al. (2008) | | Merge mother and father PTSD for Paediatric Index of Mortality Scale - 2 | 2 | 0.00 | 247 | 0 | 0 |
|  |  |  | Coakley et al. (2010) | | Medical Records - Injury Severity Score (Anatomical Scoring) | 1 | -0.09 | 51 |  |  |
|  |  |  | De Vries et al. (1999) | | Injury Severity Score (Medical Notes) | 1 | 0.0 | 102 |  |  |
|  |  |  | De Young et al. (2014) | | Burn Size - Total Body Surface Area | 1 | 0.30 | 120 |  |  |
|  |  |  | Egberts et al. (2016/2016)/ Pan et al. (2015) | | Merge mother and father PTSD for Burn Size - Total Body Surface Area | 2 | -0.04 | 162 | -0.19 | 0.09 |
|  |  |  | Hall et al. (2006) | | Burn Size - Total Body Surface Area | 1 | 0.42 | 62 |  |  |
|  |  |  | Kassam-Adams et al. (2009) | | Injury Severity Score (Medial Records) | 1 | 0.20 | 251 |  |  |
|  |  |  | Kassam-Adams et al. (2015) | | Injury Severity Score (Medical Notes) | 1 | 0.00 | 178 |  |  |
|  |  |  | LeDoux et al. (1998) | | Burn Size - Total Body Surface Area | 1 | 0.00 | 35 |  |  |
|  |  |  | Lefkowitz et al. (2010) | | NICU Medical Severity Rating | 1 | -0.11 | 85 |  |  |
|  |  |  | Martin-Herz et al. (2012) | | AIS/Injury Severity Score (Medical Notes) | 1 | 0.02 | 92 |  |  |
|  |  |  | Meiser-Stedman et al. (2017)/Hiller et al. (2016) | | Triage Rating of Trauma Severity | 1 | 0.06 | 56 |  |  |
|  |  |  | Rees et al. (2004) | | Medical Records - Injury Severity Score | 1 | 0.49 | 60 |  |  |
|  |  |  | Rizzone et al. (1994) | | Burn Size - Total Body Surface Area | 1 | 0.42 | 25 |  |  |
|  |  |  | Rodriguez-Rey & Alsonso-Tapia (2017) | | Paediatric Index of Mortality Scale - 2 | 1 | -0.05 | 143 |  |  |
|  |  |  | Willebrand & Sveen (2016/2016) | | Merge Burn Size - Total Burn Surface Area and Total Burn Surface Area - Full thickness burn | 2 | 0.24 | 106 | 0.22 | 0.26 |
|  |  | Hospital Admission | | | | | | | | |
|  |  |  | De Vries et al. (1999) | | Medical Records | 1 | 0.03 | 102 |  |  |
|  |  |  | Kassam-Adams et al. (2015) | | Medical Records | 1 | 0.00 | 178 |  |  |
|  |  |  | Sturms et al. (2005) | | Medical Records | 1 | 0.31 | 79 |  |  |
|  |  | Length of Hospital Admission | | | | | | | | |
|  |  |  | Balluffi et al. (2004) | | Medical Records | 1 | 0.00 | 161 |  |  |
|  |  |  | Bronner et al. (2008) | | Merge mother and father PTSD for Medical Records | 2 | 0.00 | 247 | 0 | 0 |
|  |  |  | Chang et al. (2016) | | Medical Records | 1 | 0.04 | 102 |  |  |
|  |  |  | Franck et al. (2015) | | Medical Records | 1 | 0.25 | 107 |  |  |
|  |  |  | Landolt et al. (1998) | | Medical Records | 1 | 0.34 | 29 |  |  |
|  |  |  | Landolt et al. (2003) | | Merge mother and father PTSD for Medical Records | 2 | 0.30 | 355 | 0.26 | 0.34 |
|  |  |  | Lefkowitz et al. (2010) | | Medical Records | 1 | -0.13 | 85 |  |  |
|  |  |  | Rees et al. (2004) | | Medical Records | 1 | 0.49 | 60 |  |  |
|  |  |  | Willebrand & Sveen (2016/2016) | | Medical Records | 1 | 0.23 | 106 |  |  |
|  |  | Parent direct exposure to trauma | | | | | | | | |
|  |  |  | Allenou et al. (2010) | | Parent was co-victim or witnessed the trauma | 1 | 0.49 | 72 |  |  |
|  |  |  | Bryant et al. (2004) | | Parent involved in or witnessed the trauma | 1 | 0.00 | 80 |  |  |
|  |  |  | De Viries et al. (1999) | | Standard Demographic Information | 1 | 0.51 | 102 |  |  |
|  |  |  | Kassam-Adams et al. (2009) | | Standard Demographic Information | 1 | 0.04 | 251 |  |  |
|  |  |  | Meiser-Stedman et al. (2017)/Hiller et al. (2016) | | Standard Demographic Information | 1 | 0.18 | 56 |  |  |
|  |  |  | Rizzone et al. (1994) | | Proximity between child and parent at time of burn | 1 | -0.28 | 25 |  |  |
|  |  |  | Winston et al. (2003) | | Parent witness the trauma | 1 | 0.07 | 162 |  |  |
| Parent Factors | | | | | | | | | | |
|  | Parent pre-trauma characteristics | | | | | | | | | |
|  |  | Older Parent Age | | | | | | | | |
|  |  |  | Chang et al. (2016) | | Standard Demographic Information | 1 | 0.08 | 102 |  |  |
|  |  |  | Lefkowitz et al. (2010) | | Standard Demographic Information | 1 | 0.07 | 85 |  |  |
|  |  |  | Martin-Herz et al. (2012) | | Standard Demographic Information | 1 | 0.01 | 92 |  |  |
|  |  | Female Gender | | | | | | | | |
|  |  |  | Balluffi et al. (2004) | | Standard Demographic Information | 1 | 0.47 | 161 |  |  |
|  |  |  | Binder et al. (2011) | | Standard Demographic Information | 1 | 0.30 | 40 |  |  |
|  |  |  | Bronner et al. (2008) | | Standard Demographic Information | 1 | 0.15 | 247 |  |  |
|  |  |  | Franck et al. (2015) | | Standard Demographic Information | 1 | 0.00 | 107 |  |  |
|  |  |  | Kassam-Adams et al. (2009) | | Standard Demographic Information | 1 | 0.06 | 251 |  |  |
|  |  |  | Kassam-Adams et al. (2015) | | Standard Demographic Information | 1 | 0.21 | 178 |  |  |
|  |  |  | Landolt et al. (2012) | | Standard Demographic Information | 1 | 0.22 | 460 |  |  |
|  |  |  | Martin-Herz et al. (2012) | | Standard Demographic Information | 1 | -0.26 | 92 |  |  |
|  |  | Race (BME) | | | | | | | | |
|  |  |  | Balluffi et al. (2004) | | Standard Demographic Information | 1 | 0.28 | 161 |  |  |
|  |  |  | Coakley et al. (2010) | | Standard Demographic Information | 1 | 0.27 | 51 |  |  |
|  |  |  | Franck et al. (2015) | | Standard Demographic Information | 1 | 0.00 | 107 |  |  |
|  |  |  | Kassam-Adams et al. (2009) | | Standard Demographic Information | 1 | 0.23 | 251 |  |  |
|  |  |  | Lefkowitz et al. (2010) | | Standard Demographic Information | 1 | -0.17 | 85 |  |  |
|  |  |  | Martin-Herz et al. (2012) | | Standard Demographic Information | 1 | 0.48 | 92 |  |  |
|  |  | Low SES | | | | | | | | |
|  |  |  | Chang et al. (2016) | | Standard Demographic Information - merge Unemployment, education level and low income | 3 | 0.05 | 102 | 0 | 0.13 |
|  |  |  | Coakley et al. (2010) | | Standard Demographic Information | 1 | -0.21 | 51 |  |  |
|  |  |  | Franck et al. (2015) | | Standard Demographic Information – merge unemployment, education level and low SES | 3 | 0.00 | 107 | 0 | 0 |
|  |  |  | Kassam-Adams et al. (2009) | | Standard Demographic Information | 1 | -0.20 | 251 |  |  |
|  |  |  | Landolt et al. (2003) | | Standard Demographic Information - merge mother and father PTSD for low SES | 2 | 0.09 | 180 | 0.03 | 0.15 |
|  |  | Previous trauma or Mental Health Difficulty | | | | | | | | |
|  |  |  | Coakley et al. (2010) | | Question about exposure to previous trauma/psychopathology | 1 | 0.52 | 51 |  |  |
|  |  |  | De Young et al. (2014) | | Demographic Questionnaire | 1 | 0.25 | 120 |  |  |
|  |  |  | Franck et al. (2015) | | Question about parent’s prior hospitalisation | 1 | 0.00 | 107 |  |  |
|  |  |  | Kassam-Adams et al. (2009) | | Traumatic Events Screening Inventory | 1 | 0.30 | 251 |  |  |
|  |  |  | Landolt et al. (2003) | | Merge mother and father PTSD for number of preceding Life events - self developed Scale | 2 | 0.06 | 355 | -0.04 | 0.15 |
|  |  |  | Lefkowitz et al. (2010) | | Merge self-report history of depression, anxiety or mental illness | 3 | 0.44 | 85 | 0.38 | 0.50 |
|  |  |  | Martin-Herz et al. (2012) | | World Health Organisation Traumatic Event Inventory | 1 | 0.07 | 92 |  |  |
|  | Parent peri-trauma variables | | | | | | | | | |
|  |  | Perceived Severity of trauma | | | | | | | | |
|  |  |  | Balluffi et al. (2004) | | Parent reported worry that child might die. | 1 | 0.28 | 161 |  |  |
|  |  |  | Coakley et al. (2010) | | Rating of trauma severity | 1 | 0.61 | 51 |  |  |
|  |  |  | Kassam-Adams et al. (2009) | | Parent reported worry that child might die. | 1 | 0.30 | 251 |  |  |
|  |  |  | Lefkowitz et al. (2010) | | Parent perceived Injury Severity Score | 1 | 0.13 | 85 |  |  |
|  |  |  | Meiser-Stedman et al. (2017)/Hiller et al. (2016) | | Parent report subjective threat | 1 | 0.07 | 56 |  |  |
|  |  |  | Rees et al. (2004) | | Parent perceived injury Severity Score | 1 | 0.47 | 60 |  |  |
|  |  |  | Rodriguez-Rey & Alsonso-Tapia (2017) | | Parent perceived illness severity | 1 | 0.15 | 143 |  |  |
|  |  | Peritraumatic Dissociation | | | | | | | | |
|  |  |  | Allenou et al. (2010) | | Merge mother and father PTSD with mother and father Peritraumatic Dissociative Experiences Questionnaire | 4 | 0.20 | 100 | 0.28 | 0.37 |
|  |  |  | Hall et al. (2006) | | Stanford Acute Stress Reaction Questionnaire | 1 | 0.41 | 62 |  |  |
|  |  |  | Meiser-Stedman et al. (2017)/Hiller et al. (2016) | | Demographic Questionnaire | 1 | 0.05 | 56 |  |  |
|  | Parent post-trauma variables | | | | | | | | | |
|  |  | Acute Stress Disorder | | | | | | | | |
|  |  |  | Balluffi et al. (2004) | | Acute Stress Disorder Scale | 1 | 0.62 | 161 |  |  |
|  |  |  | Egberts et al. (2016/2016)/ Pan et al. (2015) | | Merge Mother and father PTSD with mother and father ASD scores on the Impact of Events Scale | 4 | 0.53 | 202 | 0.40 | 0.70 |
|  |  |  | Kassam-Adams et al. (2009) | | Stanford Acute Stress Reaction Questionnaire | 1 | 0.54 | 251 |  |  |
|  |  |  | Lefkowitz et al. (2010) | | Acute Stress Disorder Scale | 1 | 0.62 | 85 |  |  |
|  |  |  | Martin-Herz et al. (2012) | | PTSD Checklist | 1 | 0.03 | 92 |  |  |
|  |  | Depression | | | | | | | | |
|  |  |  | Chang et al. (2016) | | Centre for Epidemiological Studies Depression Scale | 1 | 0.52 | 102 |  |  |
|  |  |  | Franck et al. (2015) | | Hospital Anxiety and Depression Scale | 1 | 0.27 | 107 |  |  |
|  |  |  | Kassam-Adams et al. (2015) | | Centre for Epidemiological Studies Depression Scale | 1 | 0.66 | 178 |  |  |
|  |  |  | Lefkowitz et al. (2010) | | Postpartum Depression Screening Scale | 1 | 0.82 | 85 |  |  |
|  |  |  | Martin-Herz et al. (2012) | | Centre for Epidemiological Studies Depression Scale | 1 | 0.04 | 92 |  |  |
|  |  |  | Rodriguez-Rey & Alsonso-Tapia (2017) | | Hospital Anxiety and Depression Scale | 1 | 0.68 | 143 |  |  |
|  |  |  | Scheeringa et al. (2015) | | Beck Depression Inventory | 1 | 0.80 | 62 |  |  |
|  |  | Anxiety | | | | | | | | |
|  |  |  | Franck et al. (2015) | | Hospital Anxiety and Depression Scale | 1 | 0.49 | 107 |  |  |
|  |  |  | Hall et al. (2006) | | Brief Symptom Inventory | 1 | 0.48 | 62 |  |  |
|  |  |  | Meiser-Stedman et al. (2017)/Hiller et al. (2016) | | Anxiety Sensitivity Index | 1 | 0.01 | 56 |  |  |
|  |  |  | Rodriguez-Rey & Alsonso-Tapia (2017) | | Hospital Anxiety and Depression Scale | 1 | 0.67 | 143 |  |  |
|  |  | Stress | | | | | | | | |
|  |  |  | Binder et al. (2011) | | Postnatal Complications Rating - Parent Stress | 1 | 0.26 | 40 |  |  |
|  |  |  | Lefkowitz et al. (2010) | | Total number of concurrent stressors | 1 | 0.34 | 85 |  |  |
|  |  |  | Ribi et al. (2007) | | Self-report Stress Appraisal Questions’ | 1 | 0.56 | 139 |  |  |
|  |  |  | Rizzone et al. (1994) | | Parent Rating of stress at time of trauma | 1 | 0.02 | 25 |  |  |
|  |  | Psychological Distress | | | | | | | | |
|  |  |  | Allenou et al. (2010) | | Merge mother and father PTSD and mother and father Peritraumatic Distress Inventory | 4 | 0.23 | 100 | -0.01 | 0.34 |
|  |  |  | Binder et al. (2011) | | Merge mother and father PTSD for Brief Symptom Inventory | 2 | 0.00 | 40 | 0 | 0 |
|  |  |  | De Young et al. (2014) | | Parent Distress - Depression, Anxiety Stress Scale | 1 | 0.28 | 120 |  |  |
|  |  |  | Martin-Herz et al. (2012) | | Parent report 'general Mental Health' | 1 | -0.02 | 92 |  |  |
|  |  |  | Nugent et al. (2007) | | Symptom Checklist - General distress Subscale | 1 | 0.76 | 61 |  |  |
|  |  | Negative Coping Style | | | | | | | | |
|  |  |  | Franck et al. (2015) | | COPE - Negative Coping Subscale | 1 | 0.35 | 107 |  |  |
|  |  |  | Ribi et al. (2007) | | COPE - Dysfunctional Coping Subscale | 1 | 0.50 | 139 |  |  |
|  |  | Avoidance | | | | | | | | |
|  |  |  | Meiser-Stedman et al. (2017)/Hiller et al. (2016) | | Questions asked about thought suppression | 1 | 0.14 | 56 |  |  |
|  |  |  | Willebrand & Sveen (2016/2016) | | Four questions about fear-avoidance beliefs | 1 | 0.34 | 106 |  |  |
|  |  | Alcohol Use | | | | | | | | |
|  |  |  | Franck et al. (2015) | | COPE - disengagement/substance use coping subscale | 1 | 0.14 | 107 |  |  |
|  |  |  | Martin-Herz et al. (2012) | | Alcohol Use Disorders Identification Test | 1 | 0.04 | 92 |  |  |
|  |  | Sense of Blame/Guilt | | | | | | | | |
|  |  |  | De Young et al. (2014) | | COPE – Guilt Subscale | 1 | 0.28 | 120 |  |  |
|  |  |  | Meiser-Stedman et al. (2017)/Hiller et al. (2016) | | Modified version of the Posttraumatic Cognition Inventory | 1 | 0.01 | 56 |  |  |
|  |  | Neuroticism | | | | | | | | |
|  |  |  | Chang et al. (2016) | | Maudsley Personality Inventory | 1 | 0.54 | 102 |  |  |
|  |  |  | Ribi et al. (2007) | | Neo Five Factor Inventory | 1 | 0.23 | 139 |  |  |
| Child Factors | | | | | | | | | | |
|  | Child pre-trauma characteristics | | | | | | | | | |
|  |  | Younger Age | | | | | | | | |
|  |  |  | Balluffi et al. (2004) | | Standard Demographic Information | 1 | 0.00 | 161 |  |  |
|  |  |  | Bronner et al. (2008) | | Standard Demographic Information – merge mother and father PTSD | 2 | -0.01 | 247 | 0.01 | 0.02 |
|  |  |  | Coakley et al. (2010) | | Standard Demographic Information | 1 | -0.27 | 51 |  |  |
|  |  |  | De Vries et al. (1999) | | Standard Demographic Information | 1 | -0.20 | 102 |  |  |
|  |  |  | De Young et al. (2014) | | Standard Demographic Information | 1 | -0.12 | 120 |  |  |
|  |  |  | Egberts et al. (2016/2016)/ Pan et al. (2015) | | Standard Demographic Information - merge mother and father PTSD | 2 | -0.19 | 162 | -0.15 | -0.23 |
|  |  |  | Franck et al. (2015) | | Standard Demographic Information | 1 | 0.00 | 107 |  |  |
|  |  |  | Landolt et al. (1998) | | Standard Demographic Information | 1 | -0.07 | 29 |  |  |
|  |  |  | Landolt et al. (2003) | | Standard Demographic Information - merge mother and father PTSD | 2 | 0.01 | 355 | -0.02 | 0.05 |
|  |  |  | Martin-Herz et al. (2012) | | Standard Demographic Information | 1 | 0.03 | 92 |  |  |
|  |  |  | Meiser-Stedman et al. (2017)/Hiller et al. (2016) | | Standard Demographic Information | 1 | 0.05 | 56 |  |  |
|  |  |  | Willebrand & Sveen (2016/2016) | | Standard Demographic Information | 1 | -0.06 | 106 |  |  |
|  |  |  | Winston et al. (2003) | | Standard Demographic Information | 1 | -0.23 | 162 |  |  |
|  |  | Male Gender | | | | | | | | |
|  |  |  | Balluffi et al. (2004) | | Standard Demographic Information | 1 | 0.00 | 161 |  |  |
|  |  |  | Bronner et al. (2008) | | Standard Demographic Information - merge mother and father PTSD | 2 | -0.07 | 247 | 0.02 | 0.12 |
|  |  |  | Chang et al. (2016) | | Standard Demographic Information | 1 | 0.02 | 102 |  |  |
|  |  |  | Coakley et al. (2010) | | Standard Demographic Information | 1 | 0.14 | 51 |  |  |
|  |  |  | De Vries et al. (1999) | | Standard Demographic Information | 1 | 0.09 | 102 |  |  |
|  |  |  | De Young et al. (2014) | | Standard Demographic Information | 1 | 0.04 | 120 |  |  |
|  |  |  | Franck et al. (2015) | | Standard Demographic Information | 1 | 0.00 | 107 |  |  |
|  |  |  | Landolt et al. (1998) | | Standard Demographic Information | 1 | 0.03 | 29 |  |  |
|  |  |  | Landolt et al. (2003) | | Standard Demographic Information - merge mother and father PTSD | 2 | -0.08 | 355 | -0.11 | -0.07 |
|  |  |  | Martin-Herz et al. (2012) | | Standard Demographic Information | 1 | 0.31 | 92 |  |  |
|  |  |  | Meiser-Stedman et al. (2017)/Hiller et al. (2016) | | Standard Demographic Information | 1 | 0.01 | 56 |  |  |
|  |  |  | Ostrowski et al. (2007) | | Standard Demographic Information | 1 | 0.43 | 61 |  |  |
|  |  |  | Willebrand & Sveen (2016/2016) | | Standard Demographic Information | 1 | 0.05 | 106 |  |  |
|  |  | Previous Trauma/Hospital Admission | | | | | | | | |
|  |  |  | Balluffi et al. (2004) | | Prior Hospital Admissions | 1 | 0.00 | 161 |  |  |
|  |  |  | Coakley et al. (2010)* | | Structured Parent Interview | 1 | 0.26 | 51 |  |  |
|  |  |  | De Vries et al. (1999) | | Screening Question | 1 | 0.18 | 102 |  |  |
|  |  |  | Franck et al. (2015) | | Demographic Questionnaire – merge child prior hospitalisation and child readmission to hospital | 2 | 0.22 | 107 | 0.19 | 0.24 |
|  |  |  | Kassam-Adams et al. (2009) | | Traumatic Events Screening Inventory | 1 | 0.21 | 251 |  |  |
|  |  |  | Kubota (2016) | | No. of previous hospital admissions | 1 | 0.32 | 72 |  |  |
|  |  |  | Meiser-Stedman et al. (2017)/Hiller et al. (2016) | | Demographic Questionnaire | 1 | 0.03 | 56 |  |  |
|  | Child trauma related variables | | | | | | | | | |
|  |  | Medical complications | | | | | | | | |
|  |  |  | Binder et al. (2011) | | Merge mother and father PTSD with for gestational age, and birth weight | 4 | 0.10 | 40 | -0.17 | -0.04 |
|  |  |  | Bronner et al. (2008) | | Merge mother and father PTSD with Artificial Ventilation, Circulatory Support & Neuro Blocking | 6 | 0.13 | 247 | 0.02 | 0.37 |
|  |  |  | Chang et al. (2016) | | Low birth weight | 1 | 0.16 | 102 |  |  |
|  |  |  | De Young et al. (2014) | | Number of invasive procedures | 1 | 0.32 | 120 |  |  |
|  |  |  | Sturms et al. (2005) | | Presence of a Head Injury | 1 | 0.38 | 79 |  |  |
|  |  |  | Winston et al. (2003) | | Presence of an Extremity Fracture | 1 | 0.28 | 162 |  |  |
|  | Child post-trauma variables | | | | | | | | | |
|  |  | Acute Stress Disorder | | | | | | | | |
|  |  |  | Bryant et al. (2004) | | Impact of Events Scale - Child Version | 1 | 0.00 | 80 |  |  |
|  |  |  | Kassam-Adams et al. (2009) | | Child Acute Stress Disorder Questionnaire | 1 | 0.28 | 251 |  |  |
|  |  |  | Martin-Herz et al. (2012) | | Reaction Index - Adolescent Version | 1 | 0.16 | 92 |  |  |
|  |  | Post-traumatic Stress Disorder | | | | | | | | |
|  |  |  | Bryant et al. (2004) | | Impact of Events Scale - Child Version | 1 | 0.00 | 80 |  |  |
|  |  |  | De Vries et al. (1999) | | PTSD Checklist for Children - Parent Report | 1 | 0.65 | 102 |  |  |
|  |  |  | De Young et al. (2014) | | PTSD Subscale of the Diagnostic Infant Preschool Assessment | 1 | 0.6 | 120 |  |  |
|  |  |  | Hall et al. (2006) | | Child Posttraumatic Stress Disorder Reaction Index | 1 | 0.44 | 62 |  |  |
|  |  |  | Kassam-Adams et al. (2009) | | Posttraumatic Symptom Inventory (age5-7), Clinician Administered TPSD Scale for 8-17 year olds | 1 | 0.08 | 251 |  |  |
|  |  |  | Kassam-Adams et al. (2015) | | Child PTSD Symptom Scale | 1 | 0.17 | 178 |  |  |
|  |  |  | Landolt et al. (2003) | | Merge mother and father PTSD for DSM-IV Diagnostic Interview for Children and PTSD Symptom Scale | 2 | 0.00 | 355 | -0.01 | 0.02 |
|  |  |  | Meiser-Stedman et al. (2017)/Hiller et al. (2016) | | PTSD Semi-Structure Interview and Observational Record for Infants and Young Children (IORYC)/CAPS | 1 | 0.29 | 108 |  |  |
|  |  |  | Mirzamani & Bolton (2002) | | Clinician Administered PTSD Scale | 1 | 0.45 | 37 |  |  |
|  |  |  | Nugent et al. (2007) | | Clinician Administered PTSD Scale | 1 | 0.45 | 82 |  |  |
|  |  |  | Ostrowski et al. (2007) | | Clinician Administered PTSD Scale | 1 | 0.33 | 61 |  |  |
|  |  |  | Ostrowski et al. (2011) | | Clinician Administered PTSD Scale | 1 | 0.26 | 99 |  |  |
|  |  |  | Rees et al. (2004) | | Impact of Events Scale - Child Version | 1 | 0.40 | 60 |  |  |
|  |  |  | Scheeringa et al. (2015) | | Posttraumatic Stress Disorder Semi-Structured Interview and Observational Record for Infants and Young Children (PTSD-SSI) | 1 | 0.29 | 62 |  |  |
|  |  |  | Sturms et al. (2005) | | Impact of Events Scale - Child Version | 1 | 0.65 | 50 |  |  |
|  |  | Externalising problems | | | | | | | | |
|  |  |  | De Young et al. (2014) | | Child Behaviour Checklist | 1 | 0.24 | 120 |  |  |
|  |  |  | Egberts et al. (2016/2016)/ Pan et al. (2015) | | Merge mother and father PTSD with mother and father reports on CBC-L | 4 | 0.11 | 162 | 0.09 | 0.13 |
|  |  |  | Kubota (2016) | | Child Behaviour Checklist | 1 | 0.37 | 72 |  |  |
|  |  |  | LeDoux et al. (1998) | | Child Behaviour Checklist | 1 | 0.00 | 35 |  |  |
|  |  |  | Winston et al. (2003) | | Posttraumatic Stress Risk Factor Form | 1 | 0.22 | 162 |  |  |
|  |  | Poorer Recovery | | | | | | | | |
|  |  |  | Balluffi et al. (2004) | | Child having another hospital Admission | 1 | -0.24 | 161 |  |  |
|  |  |  | Franck et al. (2015) | | Hospital Records - Health Status post hospitalization | 1 | -0.37 | 107 |  |  |
|  |  |  | Kassam-Adams et al. (2015) | | Single Item - Parent Rated | 1 | -0.27 | 178 |  |  |
|  |  |  | Kubota (2016) | | Kid-KIND Questionnaire - QOL | 1 | -0.28 | 72 |  |  |
|  |  |  | Landolt et al. (2003) | | Merge mother and father PTSD for Functional Status - Single Item Question | 2 | -0.24 | 355 | -0.18 | -0.30 |
|  |  |  | Ribi et al. (2007) | | Physician rated 2-point scale | 1 | -0.31 | 139 |  |  |
|  |  | Co-morbid Psychological Problem | | | | | | | | |
|  |  |  | Egberts et al. (2016/2016)/ Pan et al. (2015) | | Merge mother and father PTSD for Low Emotional Health - Burn Outcomes Questionnaire | 2 | 0.49 | 162 | 0.48 | 0.49 |
|  |  |  | Kassam-Adams et al. (2015) | | Centre for Epidemiological Studies Depression Scale | 1 | 0.11 | 178 |  |  |
|  |  |  | Martin-Herz et al. (2012) | | Centre for Epidemiological Studies Depression Scale | 1 | 0.02 | 92 |  |  |
|  |  |  | Willebrand & Sveen (2016/2016) | | Burn Outcomes Questionnaire | 1 | 0.19 | 106 |  |  |
| Family Factors | | | | | | | | | | |
|  |  | Poor Family Functioning | | | | | | | | |
|  |  |  | Coakley et al. (2010) | | Family Assessment Device | 1 | 0.41 | 51 |  |  |
|  |  |  | Egberts et al. (2016/2016)/ Pan et al. (2015) | | Merge mother and father PTSD for Burn Outcomes Questionnaire – family disruption subscale | 2 | 0.32 | 162 | 0 | 0.62 |
|  |  |  | Franck et al. (2015) | | Family Cohesion - Family Environment Scale | 1 | 0.00 | 107 |  |  |
|  |  |  | Hall et al. (2006) | | Merge family strains and part-child conflict subscales of the Family Strains Index | 2 | 0.60 | 62 | 0.47 | 0.71 |
|  |  |  | Meiser-Stedman et al. (2017)/Hiller et al. (2016) | | Family Functioning Index - Irritable Distress Subscale | 1 | 0.07 | 56 |  |  |
|  |  |  | Kubota (2016) | | Mother’s rating of satisfaction with fathers help | 1 | 0.35 | 72 |  |  |
|  |  |  | Landolt et al. (2003) | | Merge mother and father PTSD with ‘family situation’ | 2 | 0.02 | 180 | -0.11 | 0.15 |
|  |  |  | Ribi et al. (2007) | | Family Relationships Inventory | 1 | 0.02 | 139 |  |  |
|  |  | Lack of Social Support | | | | | | | | |
|  |  |  | Franck et al. (2015) | | Merge COPE – Social Support Subscale and Duke-UNC functional social support questionnaire | 2 | 0.00 | 107 | 0 | 0.01 |
|  |  |  | Rizzone et al. (1994) | | Parent rating of how helpful are friends and family? | 1 | -0.20 | 25 |  |  |
|  |  |  | Willebrand & Sveen (2016/2016) | | Parent reported perceived lack of social support | 1 | -0.13 | 106 |  |  |
|  | | | | | | | | | | |

**Supplementary Material 6:** Table detailing the Sensitivity Analyses removing high risk of bias and mixed sample studies.

| Correlates | | All studies included | | | | High risk of bias studies removed | | | | Mixed sample studies removed | | | |
| --- | --- | --- | --- | --- | --- | --- | --- | --- | --- | --- | --- | --- | --- |
|  |  | *k* | N | r | *p* | *k* | N | r | *p* | *k* | N | r | *p* |
| Objective trauma factors | |  |  |  |  |  |  |  |  |  |  |  |  |
|  | Trauma severity | 18 | 1976 | 0.10 | 0.0125 | 15 | 1876 | 0.09 | 0.0313 | 12 | 1240 | 0.12 | 0.01 |
|  | Length of admission | 9 | 1252 | 0.16 | 0.0129 |  |  |  |  | **2** | **208** | **0.14** | **0.1437** |
|  | Parent direct exposure | 7 | 748 | 0.17 | 0.0749 | **6** | **723** | **0.22** | **0.046** |  |  |  |  |
| Parent Factors | |  |  |  |  |  |  |  |  |  |  |  |  |
|  | Parent older | 3 | 279 | 0.05 | 0.40 |  |  |  |  | 2 | 194 | 0.43 | 0.558 |
|  | Female Parent | 8 | 1536 | 0.15 | 0.0287 | **7** | **1496** | **0.134** | **0.063** | **2** | **429** | **0.13** | **0.0822** |
|  | Parent Race (BME) | 6 | 747 | 0.19 | 0.03 |  |  |  |  | 3 | 394 | 0.32 | 0.004 |
|  | Parent Low SES | 5 | 691 | -0.05 | 0.5 |  |  |  |  | 3 | 404 | -0.12 | 0.1815 |
|  | Parent Prev. trauma | 7 | 1061 | 0.23 | 0.001 |  |  |  |  | 4 | 514 | 0.28 | 0.004 |
|  | Parent perceived trauma severity | 7 | 807 | 0.29 | <0.001 |  |  |  |  | 3 | 358 | 0.34 | 0.018 |
|  | Parent Acute Stress Disorder | 5 | 791 | 0.49 | <0.001 |  |  |  |  | 3 | 545 | 0.40 | 0.0076 |
|  | Parent Depression | 7 | 769 | 0.59 | <0.001 |  |  |  |  | 4 | 434 | 0.55 | 0.020 |
|  | Parent Anxiety | 4 | 368 | 0.45 | 0.0026 |  |  |  |  | **2** | **118** | **0.26** | **0.3037** |
|  | Parent Stress | 4 | 289 | 0.35 | 0.0035 | 2 | 224 | 0.46 | 0.003 | 0* |  |  |  |
|  | Parent Psychological Distress | 5 | 413 | 0.29 | 0.0687 | 4 | 373 | 0.35 | 0.0524 | 4 | 373 | 0.35 | 0.0524 |
|  | Parent Negative Coping Style | 2 | 246 | 0.43 | <0.001 |  |  |  |  | 0* |  |  |  |
| Child Factors | |  |  |  |  |  |  |  |  |  |  |  |  |
|  | Child Younger Age | 13 | 1750 | -0.08 | 0.0128 |  |  |  |  | 8 | 851 | -0.14 | 0.003 |
|  | Child Male Gender | 13 | 1589 | 0.07 | 0.0375 |  |  |  |  | 8 | 690 | 0.13 | 0.0126 |
|  | Child previous Trauma/Hospital Admission | 7 | 800 | 0.17 | <0.001 |  |  |  |  | 4 | 460 | 0.12 | <0.001 |
|  | Child Medical complications | 6 | 750 | 0.23 | <0.001 | 5 | 710 | 0.24 | <0.001 | 4 | 463 | 0.28 | <0.001 |
|  | Child Post-traumatic Stress Disorder | 15 | 1707 | 0.36 | <0.001 |  |  |  |  | 13 | 1292 | 0.37 | <0.001 |
|  | Child Externalising problems | 5 | 551 | 0.20 | <0.001 | 4 | 516 | 0.22 | <0.001 | 4 | 479 | 0.17 | 0.001 |
|  | Child Poorer Recovery | 6 | 1012 | 0.27 | <0.001 |  |  |  |  | 1* |  |  |  |
| Family Factors | |  |  |  |  |  |  |  |  |  |  |  |  |
|  | Poor Family Functioning | 8 | 829 | 0.23 | 0.0057 |  |  |  |  | 4 | 331 | 0.36 | 0.0012 |
|  | Lack of Social Support | 3 | 238 | -0.08 | 0.2241 | 2 | 213 | -0.07 | 0.329 | 1* |  |  |  |
| *Note.* *Risk factors could no longer be meta-analyses due to too few studies. Numbers in bold highlight the risk factor estimates where the sensitivity analysis changed the significance value. | | | | | | | | | | | | | |
